# Single‐cell landscape analysis reveals systematic senescence in mammalian Down syndrome

**DOI:** 10.1002/ctm2.1310

**Published:** 2023-07-17

**Authors:** Yao Chen, Yanyu Xiao, Yanye Zhang, Renying Wang, Feixia Wang, Huajing Gao, Yifeng Liu, Runju Zhang, Huiyu Sun, Ziming Zhou, Siwen Wang, Kai Chen, Yixi Sun, Mixue Tu, Jingyi Li, Qiong Luo, Yiqing Wu, Linling Zhu, Yun Huang, Xiao Sun, Guoji Guo, Dan Zhang

**Affiliations:** ^1^ Key Laboratory of Reproductive Genetics (Ministry of Education) Department of Reproductive Endocrinology, Women's Hospital Zhejiang University School of Medicine Hangzhou China; ^2^ Center for Stem Cell and Regenerative Medicine Zhejiang University School of Medicine Hangzhou China; ^3^ Department of Reproductive Genetics Women's Hospital, Zhejiang University School of Medicine Hangzhou China; ^4^ Birth Defect Control and Prevention Research Center of Zhejiang Province Hangzhou China; ^5^ Department of Gynecology Hangzhou Women's Hospital Hangzhou China

**Keywords:** Down syndrome, fetal, senescence, single‐cell RNA sequencing, systematic analysis

## Abstract

**Background:**

Down syndrome (DS), which is characterized by various malfunctions, is the most common chromosomal disorder. As the DS population continues to grow and most of those with DS live beyond puberty, early‐onset health problems have become apparent. However, the cellular landscape and molecular alterations have not been thoroughly studied.

**Methods:**

This study utilized single‐cell resolution techniques to examine DS in humans and mice, spanning seven distinct organs. A total of 71 934 mouse and 98 207 human cells were analyzed to uncover the molecular alterations occurring in different cell types and organs related to DS, specifically starting from the fetal stage. Additionally, SA‐β‐Gal staining, western blot, and histological study were employed to verify the alterations.

**Results:**

In this study, we firstly established the transcriptomic profile of the mammalian DS, deciphering the cellular map and molecular mechanism. Our analysis indicated that DS cells across various types and organs experienced senescence stresses from as early as the fetal stage. This was marked by elevated SA‐β‐Gal activity, overexpression of cell cycle inhibitors, augmented inflammatory responses, and a loss of cellular identity. Furthermore, we found evidence of mitochondrial disturbance, an increase in ribosomal protein transcription, and heightened apoptosis in fetal DS cells. This investigation also unearthed a regulatory network driven by an HSA21 gene, which leads to genome‐wide expression changes.

**Conclusion:**

The findings from this study offer significant insights into the molecular alterations that occur in DS, shedding light on the pathological processes underlying this disorder. These results can potentially guide future research and treatment development for DS.

## 1 INTRODUCTION

Trisomy 21 (T21), the clinical term for Down syndrome (DS), is the most common survivable human chromosomal abnormality.^1^ Although prenatal diagnosis technologies have made great progress, 1 baby with DS is born per 779–1023 live births worldwide due to individual choice or other objective reasons.^2^, ^3^ Moreover, the average life expectancy with DS has increased substantially due to advanced medical treatments and improved living standards, rising from 25 years in 1983 to 60 years in 2020.^4^ The lifetime prevalence of DS continues to increase substantially and is estimated to range from 3.3–6.0 per 10 000 individuals worldwide.^2^ The increased life expectancy, unfortunately, is not paralleled by an increased health span. To provide high‐quality care to large numbers of existing and future DS patients, molecular surveys should be performed to better understand the pathological changes in DS.

Individuals with DS exhibit a unique set of symptoms and manifestations that affect multiple systems in the body, with variations in presentation among individuals.^5^ The most concerning symptom of DS is neuronal dysfunction.^6^, ^7^ The other wide‐ranging features have not been well studied, including digestive abnormalities, underactive thyroid glands, autoimmune disorders, infertility and even hearing and vision problems.^5^ However, with lifespan extension, age‐associated disorders in DS patients have become increasingly prominent.^8^ DS patients typically experience issues in the pediatric stage, and a growing number of them are now facing geriatric problems. One of the most typical features is early‐onset dementia. By the age of 40, some DS patients develop amyloid plaques and neurofibrillary tangles, which are typical characteristics of Alzheimer's disease brain pathology.^9^ The prevalence continues to increase and even reaches 90%–100% for 65‐ to 70‐year‐olds.^10^ Many other age‐associated characteristics display early onset in DS patients,^8^ including sensorineural hearing loss,^11^ osteoporosis,^12^ immune system deterioration,^13^ and epidermal thickening.^14^ Some research groups have reported cellular senescence in certain cell types or tissues in DS fetuses, primarily fibroblasts and nerve tissue,^15^, ^16^, ^17^ and lower birth weight of DS newborns.^18^ One recent study revealed that DS‐originated neural progenitor cells exhibited characteristics of senescence that could be alleviated by the application of senolytic agents.^19^ Within this framework, we hypothesized that systematic pathological alterations accumulate during fetal development, resulting in developmentally compromised cellular functions and early‐onset ageing.

Previous studies have focused mainly on the DS neuronal system and have aimed to elucidate the relationships between individual organ/tissue phenotypes and the corresponding HSA21 gene/genes. Few HSA21 genes have been implicated in the development of specific phenotypes like *APP* and *DYRK1A* in dementia,^20^ *GATA1* in hematopoietic malignancies^21^ and *DSAM* and *COL6A2* in heart disease.^22^ Other features within the spectrum await further elucidation. The HSA21 gene/genes have yet to be used to explain the wide variety of clinical phenotypes and their early onset. In addition, most of the prior studies have employed bulk RNA sequencing; thus, the findings reflect the average gene expression but are not sufficient to reveal cell‐type‐specific alterations contributing to DS.

Given the speculation that various clinical presentations and their premature features originated from systematic developmental alterations,^2^, ^5^, ^13^, ^23^, ^24^ the lack of comprehensive sampling and the limitations of bulk sequencing have limited the understanding of DS. In the current study, we aimed to build a single‐cell landscape of mammalian DS at the intrauterine stage. We reasoned that the resulting single‐cell resolution data could help elucidate the molecular alterations in the early phase of DS development more comprehensively and more precisely than prior data. Ultimately, we intended to provide new insights for physicians and DS families to improve the medical support and quality of life of DS patients. We applied Microwell‐seq^25^ to a well‐characterized DS mouse model (Ts65Dn)^26^ and specimens from human T21 fetuses to construct the transcriptional landscapes of both mouse and human DS fetuses. By systematically characterizing the transcriptional changes and cellular heterogeneity by cell type, we found signatures of several senescence stresses in both mouse and human DS. These genome‐wide molecular alterations were driven by an HSA21 gene‐derived regulatory network.

## 2 RESULTS

**FIGURE 1:**
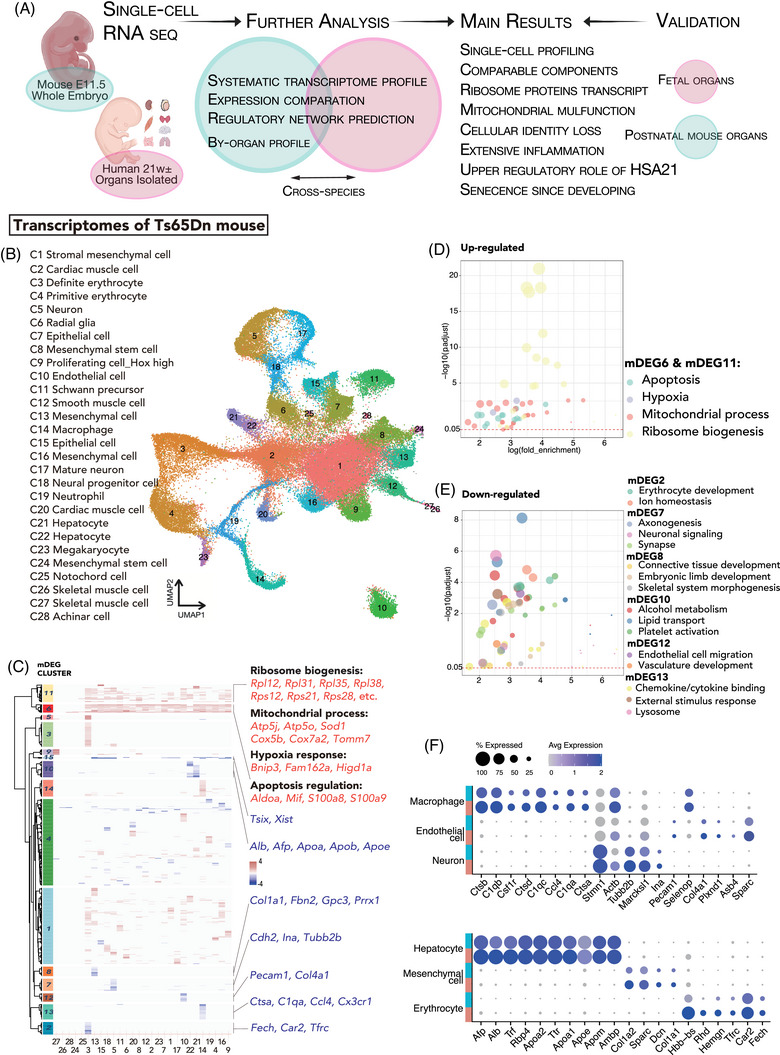
Single‐cell transcriptomes of the Ts65Dn mouse model of Down syndrome (DS). (A) Single‐cell RNA‐seq workflow. (B) Uniform manifold approximation and projection (UMAP) presentation of combined DS and normal cells. The cell clusters are labelled in different colours. The annotated cluster identities are listed on the left. (C) Hierarchical clustering of the log fold changes of mouse differentially expressed genes (mDEGs) between DS and normal subclusters for each cell cluster. The resulting clusters were labelled mDEG1‐15 on the left plots. (D, E) Bubble plot showing the main GO terms enriched in mDEG clusters. (F) Dot plot showing the scaled average expression level of functional genes and marker genes in certain mouse cell clusters split by karyotype.

We sampled a DS mouse model at embryonic day (E) 11.5 during organogenesis and enzymatically digested each whole‐mount embryo. We collected human samples at approximately 22 weeks of gestation, subjected to their amniocentesis timing. Considering the scale and the cell counts of organs within the second trimester, we digested the human samples by organ. We successively profiled mouse and human DS and subjected the samples to systematic analysis and cross‐species analysis (Figure 1A). The main findings from these two datasets corroborated each other.

### 2.1 Single‐cell transcriptomic profiling of a mouse DS model

Organogenesis is a remarkable process in which cells from different germ layers transform into embryos to form complex organisms. Specifically, a mouse embryo expands from hundreds of thousands of cells to over ten million cells during the period from E9.5 to E13.5, concurrently developing nearly all major organ systems.^27^ To systematically survey the molecular alterations that occur during DS organogenesis at single‐cell resolution, we obtained six Ts65Dn mouse fetuses at E11.5. Mouse tail biopsies were collected for genotyping. We identified three mouse fetuses with trisomic distal chromosome 16 (Ts65Dn mouse model for DS, referred to as the DS group hereafter) and three paired littermate controls (referred to as the normal group). Each whole mouse fetus was digested to produce single‐cell suspensions. We performed 3′ droplet‐based single‐cell RNA sequencing (Microwell‐seq) and produced 71 934 cells, which passed the standard quality control dataset filter and were retained for downstream analysis. Despite relatively shallow sequencing, we obtained a mean of 945 unique molecular identifiers (UMIs) and 592 genes per cell from mouse fetuses.

We successively constructed mouse and human cell atlases with Microwell‐seq.^25^, ^28^ However, Microwell‐seq is not as widespread as other single‐cell sequencing platforms. To further verify the data authenticity, we subjected our data of diploid mouse embryos together with the published E11.5 dataset to uniform manifold approximation and projection (UMAP) (Figure S1A,B).^27^ Cells from both datasets shared a similar distribution pattern, indicating that the capture efficiency of Microwell‐seq was reliable and that it was feasible to use Microwell‐seq to assess the impact of DS.

### 2.2 DS results in clinical phenotypes by modifying molecular and cellular functions rather than altering tissue composition

We first conducted UMAP analysis on the combined trisomic and disomic mouse datasets with the Seurat package.^29^ A total of 28 clusters were identified and classified into seven distinct lineages according to their identities, including stromal, epithelial, hemopoietic, immune, muscular, hepatic and neuronal lineages (Figure 1B and Figure S1C). We manually annotated all 28 clusters by investigating the expression patterns of known marker genes for fibroblasts (marked by *Col3a1*), mesenchymal cells (*Spark*), epithelial cells (*Epcam*), endothelial cells (*Esam*), erythroid cells (*Hbb*‐*bs*, *Gata1*), megakaryocytes (*Pf4*), macrophages (*C1qb*), neutrophils (*S100a8*), cardiac muscle cells (*Myl2*), skeletal muscle cells (*Tnnc1*), smooth muscle cells (*Acta2*), hepatocytes (*Afp*, *Alb*), neurons (*Nefl*) and radial glia (*Fabp7*) (Figure S1D). The detailed marker genes for each cluster are listed in Table S1.

In general, after the removal of the batch effect, DS cells and normal cells matched well (Figure S1E,F). Each of the clusters comprised comparable proportions of cells from the DS and normal groups. No DS‐ or normal‐specific cell clusters were identified, suggesting that DS produces clinical phenotypes by altering molecular and cellular functions rather than tissue composition. Notably, by evaluating the expression of the male and female RNA markers^30^ in each of the embryo samples, we inferred the sample sex. Only one of the three normal embryos was identified as female, while the other two normal and all three DS embryos were identified as male (Figure S1G).

### 2.3 Systematic differential gene expression analysis

We compared the gene expression levels in DS and normal mice within each cell type and identified a total of 1877 differentially expressed genes (DEGs, in order to distinguish the mouse and human DEGs discussed later in the text, mouse DEGs would be referred to as mDEGs, and hDEGs for human data) (Table S2). The predominance of upregulated genes suggested that these genes were part of an activated signature in DS cells, which was in line with our expectations (Figure S1H). Since human T21 and its corresponding mouse model feature extra HSA21 gene copies, it was not surprising that many genes were overexpressed. Similar to related studies,^23^, ^31^ our study revealed that only ∼1.2% of mDEGs were HSA21 homologous genes (padjD < 0.05), 50% of which had expression at least 2‐fold higher in DS samples than in normal samples. One unexpected result was that within all cell types, bunches of ribosomal protein‐coding genes (RPs) were upregulated significantly (Figure S1H). In over 10% of DS and normal cells, 8.08% and 6.77% of the genes detected were RPs, respectively, while 16% of the DEGs were RPs. Generally, these findings revealed an activated signature and the genome‐wide transcriptional disruption of DS cells at single‐cell resolution.

### 2.4 Single‐cell systematic analysis reveals prominent features of DS cells

We hierarchically clustered the DEGs to summarize the coordinated gene expression changes in each cell type. The coordinated changes could be divided into clusters of upregulated or downregulated DEGs in a specific cell type. All of the DEGs between DS and normal mouse fetuses were divided into 15 DEG clusters revealing cell‐type‐specific or shared features (Figure 1C and Table S3). For instance, both the DEG6 and DEG11 clusters comprised highly and coordinately upregulated genes across most cell types. They were enriched for mitochondrial processes (*Atp5j, Atp5o, Cox5b* and *Cox7a2*) and apoptosis (*Bnip3, Mif, S100a8* and *Rps27l*), consistent with the findings of previous studies on DS fibroblasts, neuroblasts, and iPSCs.^32^, ^33^, ^34^, ^35^ Mitochondrial dysfunctions and the hypoxia‐related pathways enriched in the DEG11 cluster have long been associated with DS and are even considered therapeutic targets. Our analysis highlighted mitochondrial alteration as a shared feature across most cell types in DS individuals, not only fibroblasts and neuroblasts.

### 2.5 Active transcription of ribosomal proteins

The DEG11 cluster was enriched with ribosomal process‐related DEGs owing to the significant and widespread upregulation of RPs (Figure 1D). This is consistent with the proteome profile of T21 fibroblasts.^36^ These pathogenic features are somewhat counterintuitive. Given that ribosome biogenesis is one of the most energetically demanding and metabolically active processes in the cell,^37^, ^38^ the extension of the transcriptional activity of ribosomal proteins and the impairment of mitochondrial biological processes seem contradictory to each other. These changes may leave the cell in a vicious cycle, causing and worsening cell dysfunction and even initiating proapoptotic signalling.

### 2.6 Mitochondrial malfunction

Mitochondria power cell life processes and play critical roles in cell death.^39^ In response to oxidative and energy stresses, the mitochondrial volume changes. Malfunctions of respiration and overload of byproducts change mitochondrial membrane permeability, which further compromises the bioenergetic function and structural integrity of mitochondria. However, under continuing pathophysiological conditions, the ion homeostasis of the matrix changes, which leads to mitochondrial swelling.^40^ In the DEG6 cluster, mitochondrial processes were coordinately upregulated among most cell types (Figure 1C,D). We examined mitochondrial morphology using transmission electron microscopy (TEM) and observed more damaged mitochondria in DS muscle cells with decreasing cristae area and comparable maximal cristae width (Figure S2A–E). The mitochondrial matrix in DS cells presented decreased electron density with multifocal vacuolar degeneration and a disordered arrangement of mitochondrial cristae. Moderate increases in mitochondrial matrix volume can stimulate mitochondrial function and metabolism (gluconeogenesis, electron transport chain activity), representing an adaptive response.^32^, ^41^, ^42^ Excessive mitochondrial swelling is a central event that induces cell death through apoptosis or necrosis depending on the availability of ATP.^43^ We thus estimated the expression of mitochondrial functional genes in mouse muscle cells using quantitative polymerase chain reaction (qPCR). Genes related to oxidative phosphorylation (*Atp5a1*, *Atp5b1* and *Atp5c1*), mitochondrial fission and fusion (*Mfn1* and *Mfn2*), and mitochondrial transport (*Macf1*) were significantly upregulated in DS muscle cells (Figure S2F). Western Blot (WB) analysis also indicated upregulated expression of the apoptosis‐inducing factor (Aif) in DS muscle cells (Figure S2G), which induces caspase‐dependent cell death in response to the change of mitochondrial membrane potential.^44^ Prolonged alteration of mitochondrial homeostasis acts as a central nexus among different cell death modalities.^45^ Changes in mitochondrial volume have been linked to a variety of critical biological processes and diseases.^46^ These morphological and functional alterations during the early life of individuals with DS might further accumulate or be magnified in later development.

We next examined the enrichment of DEGs in apoptosis‐related pathways using immunohistochemical TUNEL staining. Semiquantitative analysis indicated that TUNEL signalling was increased, indicating apoptosis, in the intestine (*p* < .05) and testes (*p* < .05) in the DS mouse model. Due to limitations in sample size, we stained pairs of 3‐month‐old mouse tissues, while we sequenced mouse fetuses. However, the differences between groups were significant, indicating variation to a certain extent. The overexpression of RPs and the enrichment of ribosome‐related pathways were also tested by immunohistochemical staining and semi‐quantified. We used Rps3 as an example, whose expression was significantly increased in the DS intestine, skeletal muscle, and testes (*p* < .05) (Figure S4).

### 2.7 Cellular identity loss across different cell types

We also summarized clusters of genes that were coordinately downregulated within several cell types with specialized functions. In definite erythroid cells (DEG2 cluster) in DS, the downregulated genes were enriched in ion homeostasis and erythrocyte development. In DS endothelial cells, downregulated genes (DEG12 cluster) were involved in vasculature development and endothelial cell migration, including *Pecam1*, *Card10*, *Col4a1* and *Plxnd1*. Downregulated genes in hepatocytes (DEG10 cluster) were enriched in lipid transport, alcohol metabolism, and platelet activation; these genes included *Alb*, *Afp*, *Apoa*, *Apob*, etc. DEGs in mesenchymal cells (DEG8 cluster), including *Col1a1*, *Fbn2*, *Gpc3*, and *Msx2*, were enriched in functions related to embryonic limb, bone, and connective tissue development. Within the immune system, downregulated genes in macrophages (DEG13 cluster) were enriched in the lysosome, external stimulus‐response, and chemokine/cytokine binding. The downregulated genes in DS neurons (DEG7 cluster) were related to synapses, axonogenesis, and neuronal signalling (*Actb*, *Ina*, *Tubb2b*, *Cdh2* and *Rab11b*). We visualized the expression levels of these specialized functional genes or cell fate‐determining genes (Figure 1F). The findings were compatible with those of previous studies on the reduced capacity of DS neurons to form functional neural networks.^23^ It was a rather interesting outcome that genes and pathways that determine cell‐specialized functions were downregulated in DS fetuses in most of the cell types. It can thus be speculated that the bulk of DS cells are not sufficiently able to exert their specialized functions. DS cells may therefore be “below grade”. There is, however, another possible explanation. Disrupted expression of cell‐type defining features is often associated with ageing. Relatively old cells or tissues are characterized by an immature cellular fate,^47^ cellular identity loss,^48^, ^49^ and dedifferentiation.^50^ The extended cellular identity losses in different DS tissues and cell types suggest that ageing occurs in DS cells. Our single‐cell‐level dissection of the DS mouse model highlighted hyperactive RP transcription, mitochondrial malfunction, enhanced apoptosis, and diminished cellular identity in DS cells.

### 2.8 Human DS fetus and Ts65Dn gene signatures share a similar trend

#### 2.8.1 Single‐cell transcriptomic profiling of human DS

**FIGURE 2:**
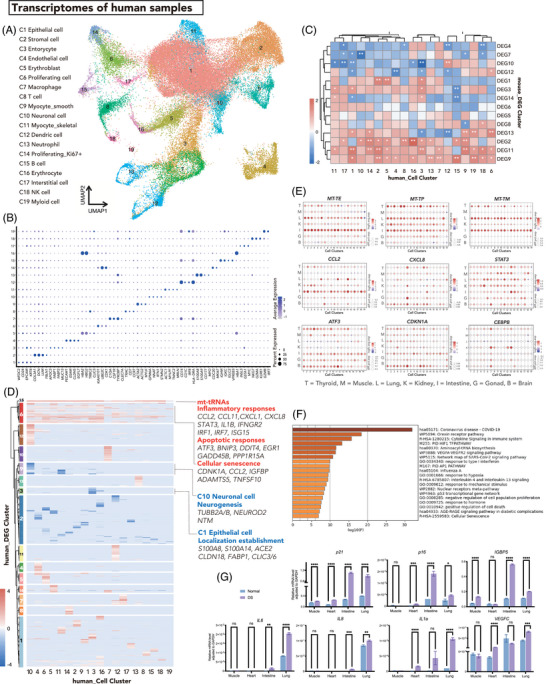
Single‐cell transcriptomes of human Down syndrome (DS) fetuses. (A) Uniform manifold approximation and projection (UMAP) presentation of combined human DS and normal cells. The cell clusters are labelled in different colours. The annotated cluster identities are listed on the left. (B) Dot plot showing the expression patterns of selected markers of each human cell cluster. (C) Enrichment of mouse differentially expressed gene (DEG) clusters onto different human cell clusters. The shades of colour show the enrichment scores and the asterisks represent the significance of the enrichment. (D) Hierarchical clustering of the log fold change of human DEGs (hDEGs) between DS and normal subclusters for each human cell cluster. The resulting clusters were labelled hDEG1‐15 on the left plots. (E) Dot plot visualization of the relative expression levels of mitochondrial tRNA and mRNA in human DS cells compared to normal cells. The size of the dot encodes the percentage of cells within a cell cluster, and the colour encodes the average expression level. (F) GO enrichment of hDEG cluster 15. (G) Relative expression levels of senescence genes in different human DS and normal tissues adjusted to those of GAPDH. The expression was measured by qPCR.

To verify the key findings observed above in the mouse model, we sampled two T21 fetuses and four euploid fetuses at 20–24 weeks post‐fertilization after obtaining ethics approvals and informed consent. Chromosome karyotyping was performed with amniotic fluid samples during prenatal examinations. Seven organs were sequenced, namely, the cortex, thyroid, lung, intestine, kidney, testis, and muscle. We again performed 3′ droplet‐based Microwell‐seq and produced a total of 98 207 cells, and we sampled 55 184 cells for further analysis to ensure the cell count comparability between DS and normal (organ‐matched) samples. Unsupervised clustering revealed 19 transcriptionally distinct preclusters, which were visualized in two dimensions by UMAP (Figure 2A). We manually assigned these 19 clusters according to the expression of known cell‐type‐specific markers (Figure 2B and Table S4). DS cells were distributed comparably to normal cells (Figure S5A,B). Cells from different organs were dimensionally reduced to distribute into all 19 clusters (Figure S5C,D). Although the parameters employed for clustering were insufficient to meticulously distinguish cellular subtypes, the 19 generated preclusters enabled us to perform the following systematic analysis.

For human samples, we documented each of their sexes and verified this by evaluating the transcription of sex markers (*RPS4Y1* for males and *XIST* for females).^30^ Only the lung sample of the normal group was sampled from a female. We next plotted the well‐known sex‐bias genes with human DEGs (hDEGs) in the lung and male sample pairs of other organs and found that they overlapped randomly^51^ (Figure S5E), indicating sex bias was limited in this study. We concluded that our hDEGs and further results were not correlated to sex but that applying the results of this study to male DS would be most appropriate.

#### 2.8.2 Conserved and nonconserved features of human DS cells

We again analyzed gene expression differences between DS and normal cells within each cell cluster and generated a list of hDEGs (Table S5). We mapped each of the DEG clusters identified in the mouse dataset onto the hDEGs by cell cluster (Figure 2C). DEG11, which was extensively upregulated in most of the mouse cell clusters, showed coordinate enrichment in most human DS cell clusters. This suggests that mouse DEG11 related to ribosome biogenesis, mitochondrial processes, the hypoxia response, and apoptosis regulation was also altered in human DS fetuses. Histological verification carried out in human samples also indicated a trend (though not significant) of increased apoptosis in human DS testis and intestine samples (Figure S6A,C,D) and significant overexpression of RPS3 in human DS muscle (Figure S6B,E). Expression alterations of mitochondrial functional genes were also testified with human samples (Figure S6F).

We further examined the orthologous DEGs across human and mouse datasets (Figure S7). With 12971 mouse DEGs (hereafter mDEGs) and 14776 hDEGs inputted (Table S6), we identified only a few orthologous up‐ or downregulated mDEGs and hDEGs that exactly matched in DS cells. The gene expression patterns were more similar among different cell lineages within a species (human or mouse) than between the corresponding cell lineages of these two species. This rather low match rate might be due to the different metabolism and physiology features between humans and mice.^52^, ^53^ However, within these limited orthologous DEGs, RPs were relatively conspicuous. These findings again indicate the prominence of RP expression in DS pathology. In addition, we observed consistent downregulation of cellular identity marker genes in human and mouse datasets, such as *ELAVL3/Elavl3*, *TUBB2B/Tubb2b*, and *INA/Ina* in neuronal cells. Taken together, our data indicate that human cells share certain features with mouse cells under DS conditions, including active RP transcription and diminished cellular identity.

To further investigate the molecular alterations in human DS cells, we grouped hDEGs into hDEG clusters (Figure 2D and Table S7). Consistent with the mitochondrial dysfunction, active transcription, and apoptosis in the Ts65Dn mouse model, we observed a coordinatively upregulated hDEG6 cluster and hDEG15 cluster. Sixteen of 22 human mitochondrial tRNA (mt‐tRNA) genes were enriched in these two extensively upregulated hDEG clusters.^54^, ^55^ We then compared the expression levels of these mt‐tRNAs between the DS and normal cell‐by‐cell clusters, with tissue information tagged (Figure 2E and Figure S8). These mt‐tRNAs were significantly upregulated in DS cells, with certain tissue specificity. The mitochondrial genome encodes mt‐tRNA together with 13 mt‐mRNAs to encode the core subunits of the OXPHOS complexes. Cells regulate their transcription of mt‐tRNAs and mt‐mRNAs to satisfy their metabolic and energetic demands. The increased abundance of mt‐tRNA and mt‐rRNA in DS cells corresponds with the active transcription of RPs and reflects differences in mitochondrial number, morphology, activity, and biogenesis.

On the other hand, the hDEG6 cluster contained genes involved in inflammatory responses (*STAT3, IFNGR2, ISG15, IRF*, etc.), chemokine activity (*CXCL1, CXCL3, CXCL8* and *CXCL10*), cytokine activity (*CCL2* and *CCL11*), and apoptosis (*BNIP3, GADD45B*, etc.) (Figure 2D and Table S7). The expression levels of representative genes were compared, and the results indicated that these genes were indeed extensively upregulated in DS (Figure 2E and Figure S9). We then conducted a functional enrichment analysis of these coordinated genes. No terms were enriched for the hDEG15 cluster of mt‐tRNAs, but the top 20 terms/pathways enriched for the hDEG6 cluster were associated with inflammation, cell death, and cell senescence (Figure 2F). Senescence is a complex cellular process characterized by a permanent cell cycle arrest and the acquisition of senescence‐associated secretory phenotype (SASP), which includes the secretion of pro‐inflammatory cytokines, chemokines and extracellular matrix remodelling factors. This process is also associated with increased expression of cyclin‐dependent kinase (CDK) inhibitors, and mitochondrial dysfunction. In addition to the SASP‐associated chemokine, cytokine, and inflammatory response genes, the CDK inhibitor p21, also named *CDKN1A*, was significantly upregulated in several DS tissues (Figure 2G). Together, these findings corroborate the key alterations in the DS mouse model and suggest that inflammation and cell senescence play roles in human DS pathology.

#### 2.8.3 By‐organ DEG analysis echoes the findings of systematic analysis

To uncover the transcriptional perturbations of organ‐/tissue‐specific cell types, we subjected each of the organs to UMAP calculation and cell identity annotation. This analysis uncovered 12 clusters in the brain, 19 clusters in the thyroid, 10 clusters in the lungs, 14 clusters in the intestine, 15 clusters in the kidneys, 16 clusters in the testes, and 17 clusters in muscle (Figure S10 and Table S8). We pooled DS and normal cells together to help identify DS‐unique undefined clusters or deficient cell types. Within each of the organs, the majority of clusters contained mixtures of DS and normal cells. We captured a few DS‐unique clusters, such as ciliated cells in the lungs and tendon cells in the skeletal muscle, mostly due to the sampling variance. There were more DS immune cells in these organs.

We next investigated the gene expression changes in each cluster in different organs and performed GO enrichment analysis with cell‐type‐specific DEGs (Tables S9 and S10). To highlight the expression alterations of the organ/tissue‐specific cell types, we summarized the enriched GO terms for the organ‐specific cell types (Figure S11). As expected, DS cells exhibited extensive upregulation of genes related to cytosolic ribosomes, hypoxia, apoptosis, and ageing. DS cells also exhibited upregulation of genes involved in inflammation and immune response processes, such as cell chemotaxis, cytokine activity, and antigen presentation. DS cells were predicted to be sensitive to external stimuli, including bacteria, viruses, and temperature stimuli, that probably do not exist during development. However, the by‐organ analysis revealed the upregulation of genes related to extracellular collagen (extracellular matrix, ECM) signalling pathways and cell adhesion. Studies have demonstrated that too much collagen slows the migration of cells, including neurons and cardiac cells, during development in DS.^56^, ^57^, ^58^ An altered ECM and altered adhesion even affect tissue morphologists in different DS organs.^59^ On the other hand, organ‐specific clusters exhibited downregulation of genes related to their corresponding cellular functions. For example, DEGs downregulated in kidney tubule cells and podocytes were enriched in nephron development, fluid homeostasis, and cortisol responses. In the intestine, enterocytes exhibited downregulation of genes involved in small molecule metabolism, and Paneth cells exhibited downregulation of genes related to vacuolar lumen transport, etc. Radial cells in the DS brain exhibited downregulation of genes associated with synapse organization and cognition (Table S10).

The results of this by‐organ analysis echo the main findings of the systematic analysis above while highlighting the immune activity of DS cells and refining the cellular identity loss that occurs in organ‐specific cell types.

#### 2.8.4 Predicted transcription factor regulation of DS gene expression

To gain further insight into DS pathogenesis and relate gene expression alterations to intracellular signalling changes, we employed SCENIC,^60^ a computational method for the detection of transcription factors (TFs) and their downstream target genes, to reveal the regulatory network under DS conditions. It first infers a gene correlation network and then uses a motif‐based filtration approach to identify and keep only potential direct targets of each TF as modules, which are referred to as regulons.

#### 2.8.5 SCENIC reveals mouse DS‐specific regulons

**FIGURE 3:**
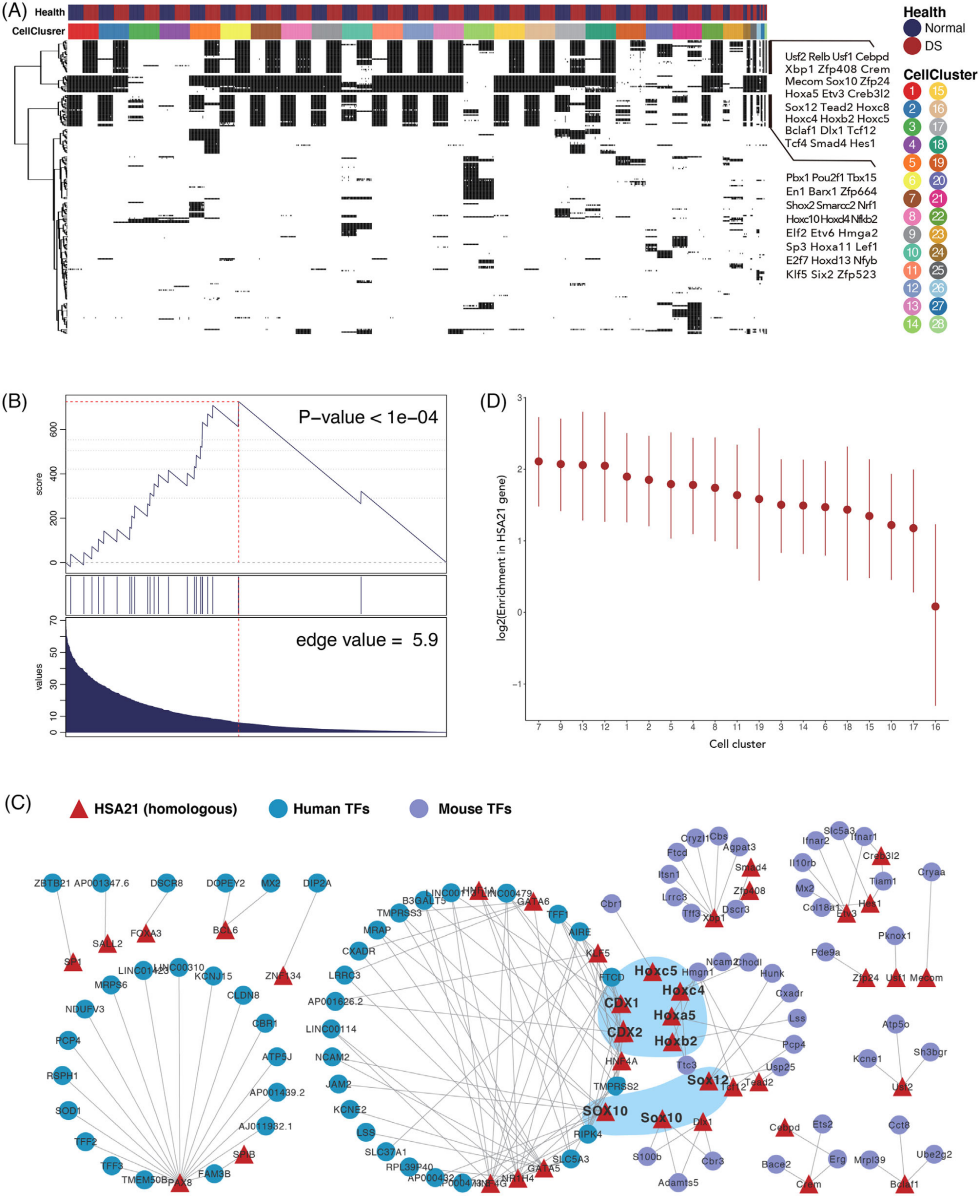
Predicted transcription factor (TF)‐mediated regulation of Down syndrome (DS) gene expression. (A) Heatmap of the binarized TF regulatory networks identified using SCENIC. (B) Gene set expression analysis (GSEA) plot for the enrichment of the TFs widely upregulated in the DS mouse model based on the hypergeometric distribution of the largest weight of links between a TF and all HSA21 genes. (C) A two‐sided Fisher's exact test was performed to estimate the enrichment of HSA21 genes with the significantly upregulated genes compared to genes located on other chromosomes. (D) Regulatory relationships identified between HSA21 (homologous) genes and active TFs.

We divided each mouse cell cluster into DS subclusters and corresponding normal subclusters. The SCENIC results suggested a very clear enrichment of a series of regulon activities in DS subclusters of almost all cell clusters (Figure 3A and Table S11). The analysis also inferred a list of downregulated regulons for most DS subclusters.

However, among these extensively enriched TFs, not a single TF was located in the triplicated region of chromosome 21. We confirmed the superiority of HSA21 (orthologous) genes in the DS course with GENIE3,^61^ which was derived to decipher genetic regulatory networks. By inferring transcriptional regulatory networks in the healthy single‐cell context, we found that the extensively upregulated TFs showed significant hypergeometric enrichment among the TFs whose expression levels were associated with HSA21 genes (Figure 3B). This result indicates the central roles of HSA21 genes in DS progression.

#### 2.8.6 Regulatory network of HSA21 genes and TFs

We then quantified the importance of HSA21 genes for the extensively enriched TFs (Figure S12A). We observed correlations between *Crem* and many of the mDEG clusters, including the extensively upregulated mDEG11 cluster. *Crem* belongs to the cAMP‐dependent TF family and is implicated in many DS‐related pathological processes, such as muscle hypertrophy and mitochondrial biogenesis.^62^ Several *Hox* genes correlated with the mDEG5 and mDEG7 clusters, consisting mainly of DEGs identified in erythroid cells and neuronal cells, respectively (Figure 2). Homeobox (HOX) genes are a family of TFs that contain a conserved DNA‐binding domain known as the homeodomain. These genes are highly conserved across species and play a crucial role in specifying cell identity during early development. Generally, overexpression of a *Hox* gene leads to stem and progenitor cell population expansion together with differentiation blockade.^63^ These impacts during fetal development can lead to leukaemia or lineage disturbances in hematopoiesis^64^, ^65^ and result in variations in neuronal populations.^66^ Moreover, *Sox10/12* play a critical role in determining cell fate and promoting differentiation in progenitor and stem cells, as well as in differentiated cells, particularly during neural crest development and neurogenesis, making them the key regulators of these processes.^67^

HSA21 genes displayed significant enrichment with disturbed TFs. We then focused on the most correlated HSA21 genes and their driven TFs (Figure 3C and Figure S12B). We noticed *Xbp1* received the most signals from HSA21 genes, while *Etv3*, *Sox12, Hes1* and *Hocx4* received signals to a lesser extent. *Hmgn1*, located on chromosome 21, provided the most driving cues. We hence propose that genes located on chromosome 21 exert dosage effects on TFs to build a DS regulatory network and result in genome‐wide gene expression perturbance.

#### 2.8.7 HSA21 gene and TF network in human DS

To verify the feasibility of this DS regulatory network in human DS, we further analyzed the regulons specifically driving DS subclusters in our human dataset. Similar to the mouse data, the human data also highlighted the regulatory roles of the SOX family and HOX genes (Figure S12C). However, more inflammatory regulators were enriched in the DS human data than in the DS mouse data, although the batch effect in the human dataset compromised the calculative power and may have limited our exploration of pathogenic regulons. HSA21 genes were also significantly enriched with TFs that were extensively upregulated (Figure 3D and Figure S12D). We next examined the regulatory network of HSA21 genes and TFs (Figure 3C). We identified eight essential mouse TFs that were homologous to human TFs (*CDX1, CDX2 and SOX10*). It is somehow unsurprising that only a limited number of TFs shown overlapped between these two species since human and mouse regulatory mechanisms are conserved to a low degree.^68^ Together, these findings confirm the DS regulatory network.

#### 2.8.8 DS cells start accumulating senescence features during the embryonic period

The features of senescent cells include inflammation, mitochondrial dysfunction, disrupted protein folding and protein transportation, and altered cell cycle.^69^, ^70^ Senescence is a stress response that limits the proliferative capacity of cells and is predominantly associated with cellular ageing. One recently published in vitro study demonstrated that DS induced senescence in iPSC‐derived neural progenitor cells. A previous study performed senescence‐associated (SA) β‐galactosidase (β‐Gal) staining and observed an approximately 15‐fold increase in the number of senescent cells among DS‐derived cells.^71^

**FIGURE 4:**
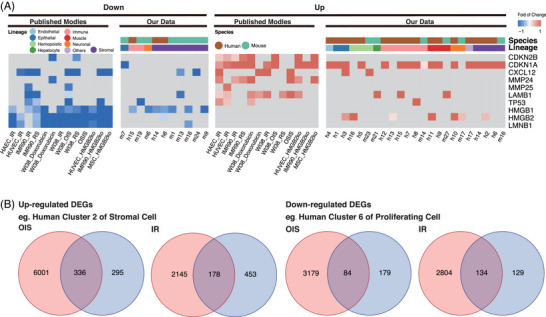
Down syndrome (DS) cells start accumulating senescence features during the embryonic period. (A) Fold changes in well‐known senescence markers in published senescence cell data and our human and mouse datasets. (B) Wayne plot showing the representative overlap of differentially expressed genes (DEGs) in senescent cells and our dataset.

To assess whether DS cells undergo senescence in vivo and in the fetal state, we examined the similarity between DS and senescent cells. We utilized transcriptomic data that was previously published in studies where senescence was induced through various methods, including oxidative stress (oxidative stress‐induced senescence [OSIS]), replicative stress (replicative senescence [RS]), ionizing radiation (IR), RAS overexpression (oncogene‐induced senescence [OIS]), and HMGB2 knockout (HMGB2ko).^19^ We evaluated the expression levels of senescence markers in our datasets. The DEGs identified in DS cells were the most correlated with the critical gene expression alterations identified in OIS, while they correlated with the changes in IR to a lesser extent (Figure 4A). DS cells exhibited marked increases in the expression of the SA cyclin‐dependent kinase inhibitor p21 (*CDNK1A*), especially in human data (Figure 2G). Western blot analysis showed that p21 expression was significantly increased in both human and mouse DS samples compared to control, (Figure S13A,B). Scattered decreases in the expression of nuclear markers of senescence (*LMNB1, P53, HMGB1* and *HMGB2*) similar to those found in senescent cells were induced through various mechanisms. We observed a significant overlap between DEGs identified in DS clusters of both human and mouse datasets and those identified in OIS and IR, respectively (Figure 4B and Table S12). We stained SA‐β‐Gal in human fetal organs and found that DS liver tissue had increased positive staining, while DS intestinal tissue showed a nonsignificant increasing trend (Figure S13C–F).

In summary, our findings suggest features of senescence are presented in DS cells even during fetal development. This includes the expression of senescence markers and a global transcriptional signature similar to that of cells undergoing OIS.

## 3 DISCUSSION

People with DS suffer from systematic pathological alterations. Previous studies have investigated individual cells, tissues, and organs but have mainly focused on neural cells and fibroblasts. In addition, recently emerged single‐cell technologies permit cellular heterogeneity dissection with genomic, epigenomic, transcriptomic and proteomic analyses. Such analyses can deepen our understanding of the molecular mechanisms underlying disease pathogeneses. We present here the first thorough single‐cell atlas of the DS mouse model and even human DS fetuses. Our systematic analysis revealed molecular features of DS at the single‐cell level. Our findings further indicate the key influences of HSA21 genes in the DS regulatory network.

### 3.1 DS ribosome stoichiometry loss

Globally, the transcription of genes encoding RPs was extensively upregulated in DS cells in both the human and mouse datasets. However, pipelines dealing with single‐cell RNA sequencing data treat RPs as markers of low‐quality cells induced by experimental procedures.^72^ The systematic overexpression of RPs detected in our datasets across different species and different cell types indicates the biological significance of RP upregulation. Ribosomal proteins are essential components of ribosomes, the molecular machines responsible for protein synthesis.^73^ These ribosomal proteins are highly expressed in rapidly dividing cells and play a critical role in cell growth and proliferation.^38^ In fact, ribosome biosynthesis can account for up to 80% of the transcriptional activity in these cells.^74^ The synthesis of these translational machinery components accounts for a significant portion of the energy expenditure in cellular life. The exact roles of these overexpressed RPs are worth further investigation. Previous studies found increased transcript levels of nonspecific RPs, including those of mitochondrial ribosomes, in old brains, leading to the formation of protein aggregates.^75^ The accumulation of misfolded proteins is particularly common in neurons due to the fact that the majority of human neurons are generated during fetal and perinatal life, and the turnover of neurons is limited in the postnatal human brain. Neurons are particularly prone to accumulate misfolded proteins. This may explain the susceptibility to early‐onset AD in the DS population. In addition, accumulated RPs are prone to deposition, and the deposits then trigger the p53 apoptosis pathway,^76^, ^77^ which was shown to be activated in our datasets. Further research is needed to identify the protein clearance system in DS cells.

### 3.2 Mitochondrial disruption and RP redundancy

Mitochondrial malfunction is another hallmark of cellular senescence and systematic ageing.^78^ The mitochondrial function of DS cells is compromised (Figure S2).^79^ Previous studies have suggested that the disorganized expression of certain HSA21 genes, such as *SOD1*, *DYRK1A*, *SUMO3* and *CBS*, contributes to the functional and structural disruption of DS mitochondria.^58^, ^80^ Mitochondria not only produce ATP but also have a complex proteomic landscape.^81^ They have a mechanism for importing and degrading misfolded proteins that are involved in cytosolic ribosomal protein regulation, which is influenced by ROS released by damaged mitochondria.^82^, ^83^ Mitochondrial stress can lead to inefficient translation termination and recycling of mitochondrial outer membrane‐associated ribosomes, causing translation stalling and ribosome‐associated quality control.

It is difficult to distinguish the prioritization and causal relationships of mitochondrial dysfunction and RP redundancy in DS cells with the current sequencing data. Further studies enrolling early‐stage DS embryos are needed. It is possible that defective mitochondria compromise cytosolic proteostasis and leave RPs aggregated. Excessive transcription of RPs, along with ageing, might overload mitochondria, leading to the structural and functional alterations we observed. However, both RP redundancy and mitochondrial dysfunction lead the cell into a vicious cycle that causes or worsens cell malfunction. These differences we identified during fetal development highlight the possibility and necessity of targeted intervention after birth. Moreover, in vitro and in vivo studies have revealed defects in the proliferation and growth of DS cells.^71^, ^84^

### 3.3 Cellular senescence during DS development

The senescence of DS cells has been recognized in recent years, specifically in detached fibroblasts and induced neural progenitors in vitro.^15^, ^19^ To our knowledge, this is the first study highlighting the occurrence of systematic senescence in different cell types across different tissues throughout DS individuals. Cellular senescence is defined as a reduction in proliferative capacity and can be induced by mitochondrial dysfunction and ROS.^85^ DS is characterized by prominent features of oxidative stress and mitochondrial dysfunction.^34^, ^86^ Extensive transcriptions of the mitochondrial genome were detected in both DS human and DS mouse samples, and morphological differences in mitochondria were also observed in this study. In this study, both CDK inhibitor (CDKN1A) and proinflammatory molecules (SASP), which are primary features of senescence, were found to be overexpressed in different DS tissues throughout the body. In addition, the compromised expression of many of the cell‐type marker genes in DS cells suggests a “blurring” of cell characteristics, which is another feature of cell ageing.^48^, ^49^ These results match the observations of senescence in earlier studies. However, we traced these clues back to the fetal stage. Our findings may help improve understanding of the early clinical features in DS individuals that emerge gradually after birth. Additionally, the tumour suppressor function of cellular senescence may explain the overall lower risk of developing solid tumour cancers in individuals with DS.^87^

Given the evidence obtained thus far, further research should be conducted to investigate the potential short‐ and long‐term impacts of senolytic drugs on DS individuals in their early stages of life.

## 4 METHODS AND MATERIALS

### 4.1 Ethics statement and tissue preparation

The sampling of fetal tissue and the research conducted in this study was approved by the Research Ethics Committee of the Women's Hospital School of Medicine of Zhejiang University (record number 20190034). Informed consent for fetal tissue collection and research was obtained from each patient/family after the decision to terminate the pregnancy legally but before the abortive procedure was performed. The T21 of fetuses was confirmed by amniocentesis, while no genetic abnormality was reported during the prenatal examination of diploid fetuses. Fetal tissues were sampled soon after delivery, transferred to the laboratory within 1 h, and immersed in 4°C PBS. The maintenance and processing of the animal model were approved by the Animal Use and Care Committee at Zhejiang University. The samples were minced into ∼1 mm pieces and enzymatically dissociated with listed enzymes in a 37°C water bath (Table S14) until microscopic examination confirmed that most of the cell clumps had dissociated into a single‐cell solution.

### 4.2 Microwell‐seq

We fabricated microwell devices and synthesized barcoded beads as described previously.^25^ Briefly, cells and beads were counted and pipetted onto microwell plates for lysis and coupling. Sequencing libraries were prepared after RNA reverse transcription, exonuclease I digestion, cDNA amplification, transposase fragmentation, and selective PCR and then sequenced by the Illumina HiSeq system.

### 4.3 Processing of Microwell‐seq data

Reads from DS and control fetuses were aligned to the *Homo sapiens* GRCh38 genome. For quality control, we filtered out cells with fewer than 500 detected transcripts. Cells with a high proportion of mitochondria‐encoded transcript counts were also excluded.

*Seurat3* ^88^ was used to perform a clustering analysis of the processed single‐cell data matrix. The digital gene expression data were log2(TPM/10+1) transformed, and the number of UMIs/gene and the percentage of mitochondrial gene content were regressed. To address differences between experimental batches and to construct a cell atlas across health states and tissues, we used Seurat3 to calculate integration anchors for batches with the function *FindIntegrationAnchors* and then performed integration with the function *IntegrateData*. The 2000 genes exhibiting the highest cell‐to‐cell variation in the dataset were subjected to initial principal component analysis. Then, the nonlinear dimensionality reduction algorithm UMAP was performed with the presumed number of principal components via the *PCElbowPlot* function and *JackStrawPlot* function. Next, we used the *FindCluster* function to cluster cells and applied the Wilcoxon rank‐sum test by running the *FindAllMarkers* function to find differentially expressed markers in each cell cluster. Finally, we referenced extensive literature to annotate each cell type and searched for specific gene expression patterns.

### 4.4 Differential expression analysis

Differential expression analysis was performed with the R package *MAST*, which fits a hurdle model to the expression of each gene.^89^ The model consists of logistic regression for the zero process (i.e. whether the gene is expressed) and linear regression for the continuous process (i.e. the expression level). To explore the disease susceptibility for different cell types and to control cell complexity, the regression model included terms to capture the effects of the cell type and the disease state on gene expression and the number of genes detected per cell. Specifically, we used the regression formula “Expi = C + D + N”, where “Expi” is the standardized log2(TP10K+1) expression vector for gene i across all cells, “C” is a binary variable reflecting cell identity, “D” is the disease state associated with cells in the cell identity, and “N” is the number of genes detected in each cell. The cells were evenly downsampled so that a maximum of 2000 cells were tested for each cell type. The model's discrete and continuous coefficients were calculated, and the *p* values were retrieved using the likelihood ratio test in *MAST*. All reported differential expression coefficients and *p* values corresponded to the discrete component of the model.

### 4.5 Functional enrichment analysis

Genes were deemed significantly differentially expressed if they had an adjusted *p*‐value less than .05 and were used for subsequent enrichment analysis. To optimize presentation, the significant DEGs of all cell types were selected to draw the heatmap and clustered with the “Ward.D2” algorithm. GO enrichment analyses were then performed on each gene cluster using the R package *clusterProfiler* with the function *enrichGO*.^90^ The GO functional enrichment results were visualized with the R package *ggplot2*.^91^

For by‐organ analysis, we enrolled clusters comprising DS cells and control cells both more than 50 and then calculated the DEGs within the cluster. DEGs with log2FC absolute value greater than 0.25 (p_val_adj < 0.05) were used for GO analysis.

### 4.6 Comparison of transcriptional alterations between human DS fetus and mouse model samples

Considering that the mouse model has a less complex genetic background than humans, we first clustered the DEGs of all mouse cell types from the above analysis into 14 groups. Then, the generated DS‐related gene sets were used as a reference to test whether human cells undergo a similar transcriptional transition in DS pathologic conditions. We used the R package *liger* to estimate enrichment of the mouse‐derived gene sets in the expression alterations of each DS human cell type based on a hypergeometric distribution.^92^

### 4.7 Confirmation of an overrepresented pattern of HSA21 genes in DS cells

To explore the potential dosage effect of HSA21 genes that may drive the transcriptional alterations in DS, we first defined the upregulated genes in each cell type with cutoffs of a coefD > 0 and a pvalD < .05 (coefficient and *p*‐value corresponding to the discrete component of the model in differential expression analysis, respectively). A two‐sided Fisher's exact test was then performed to estimate the enrichment of HSA21 genes with the significantly upregulated genes compared to genes located on other chromosomes.

### 4.8 Gene regulatory network inference

We performed gene regulatory network analysis to infer TF activity alterations in the healthy and DS cellular contexts. Pseudocells were made by aggregating single‐cell mRNA data from 20 cells in the same clusters to increase the gene number and gene expression correlation. *SCENIC* was then used with the default parameters to construct a regulatory network in both human and mouse cells.^93^ The inference was based on gene coexpression followed by motif analysis. *SCENIC* also scored the TF activity in each pseudocell with an AUCell algorithm and binarized the scores according to their normal distributions. Putative active TFs in each pseudocell were then visualized using the heatmap arranged in the order of cell clusters and under healthy conditions.

The analysis identified a group of DS‐related TFs with robustness, that was observed to be enriched only in DS cells for most cell clusters. Since HSA21 genes were observed to be enriched among DEGs and might play roles in gene expression alterations as described previously, we further hypothesized that DS‐related TFs were coregulated with HSA21 genes. The maximum coefficient of relatedness between a TF and all HSA21 genes was assessed based on the result of coexpression analysis implemented in *GENIE3*.^61^ We then tested whether the DS‐related TFs possessed a closer association with HSA21 genes than other TFs through hypergeometric enrichment for their maximum coefficient of relatedness with HSA21 genes.

### 4.9 Cross‐species analysis

First, we filtered the significant DEGs (pvalD < .05) of normal and T21 humans and mice and used *biomaRt* (version 2.4, R package) to obtain homologous genes. Then, we retained the mDEGs that were consistently upregulated or downregulated in all cell types for each mouse lineage and selected human genes with the same trends in at least one cell type of the human corresponding lineage. These human–mouse homologous genes were used as human–mouse conserved DEGs. The heatmap was drawn using *pheatmap* (version 1.0.12, R package) and showed the fold changes of DEGs in each human and mouse cell type.

### 4.10 qPCR analysis

Total RNA was extracted from the samples using the TRIzol reagent (Takara) following the manufacturer's instructions. Subsequently, the extracted RNA was reverse‐transcribed into complementary DNA (cDNA) using the reverse‐transcription kit (R233, Vazyme, China). The expression levels of the target genes were measured using SYBR Green‐based real‐time PCR assays (R711, Vazyme, China). qPCR was performed with a LightCycler 480 (Roche) system. The primer information for the gene targets is listed in Table S13. All qPCR experiments were repeated at least thrice as independent biological replicates, and results were presented as mean ± SEM.

### 4.11 Immunohistochemistry and immunofluorescence

For immunohistochemistry, 3 pairs of 3‐month‐old mice were perfused with 4% paraformaldehyde (PFA); then, the organs were dissected into 4% PFA and fixed for 2 days. To prepare the paraffin‐embedded mouse tissue sections for staining, they were deparaffinized and immersed in a retrieval solution containing 10 mM sodium citrate. The sections were then heated in an autoclave, blocked with 3% BSA, and incubated overnight with anti‐RPS3 (ab154953; Abcam), followed by anti‐HRP secondary antibodies. VECTASTAIN ABC kits and DAB peroxidase substrate kits (G1211; Servicebio) were used to perform the staining. For TUNEL staining of 10‐μm paraffin‐embedded prostate sections, an In Situ Cell Death Detection kit (11684817910; Roche) was used according to the manufacturer's instructions.

Immunofluorescence was performed on frozen sections. Human fetus organs (frozen and embedded in OTC) were sectioned at 10 μm. Frozen sections were thawed at room temperature for 10 min and washed in PBS (with 1% bovine serum albumin) three times. Slides were blocked in 5% serum at room temperature for 30 min. The sections were then incubated overnight at 4 °C with primary antibodies against RPS3 (ab154953; Abcam). After washing the sections three times with 1% BSA in PBS, the slides were incubated with secondary antibodies (Thermo Fisher) and DAPI (ab228549; Abcam) for 1 h at room temperature. TUNEL staining was performed using a one‐step TUNEL Apoptosis Detection Kit (HKI0011; Haoke). The slides were scanned using the Olympus VS200 system.

Semiquantitative analysis was performed using Fiji software (National Institutes of Health, https://fiji.sc, accessed in Dec 2022).

### 4.12 Transmission electron microscopy

Samples collected from 3‐month‐old mice (n = 3) were fixed in 2.5% glutaraldehyde at room temperature for 2 h, followed by fixation at 72°C overnight. The fixed samples were washed with PBS three times for 10 min each, then treated with 1% osmic acid for 1 h, and washed with PBS three times for 10 min each. The samples were further fixed with 2% uranyl acetate for 30 min; dehydrated using 50%, 70%, 90% and 100% ethanol for 10 min each. and washed with 100% acetone twice for 15 min each. The tissues were embedded with an embedding agent at room temperature for 2 h, transferred with the embedding agent, embedded at 37°C, and finally polymerized. Photographs were taken using a Leica UC7 microsystem and a cryo‐electron microscope (Tecnai G2 Spirit 120 kV).

Indicators of mitochondrial morphology were semi‐quantified with ImageJ software (National Institutes of Health, https://imagej.net/ij/, accessed in Dec 2022) following a previous protocol.^94^

### 4.13 SA‐β‐Gal staining

SA‐β‐Gal staining was performed using an SA‐β‐Gal staining kit (#C0602; Beyotime Institute of Biotechnology) according to the manufacturer's instructions. SA‐β‐Gal+ cells were identified as blue‐stained spots under light microscopy. Statistics were obtained from two repeat experiments using fetal samples stored at −80°C (no longer than six months). Random fields of view at equal magnification were analyzed using ImageJ software.

### 4.14 Western blot

To extract protein from human and mouse skeletal muscle, cold RIPA buffer (BL507A; Biosharp) supplemented with 1 mM phenylmethylsulfonyl fluoride (P0100; Biosharp) and protease inhibitor cocktail (HY‐K0021; MCE) was used. The protein lysates were resolved by SDS‐PAGE, and the membrane was blocked with 5% BSA before incubating with the indicated antibodies overnight at 4℃. After washing the membrane three times, it was incubated with HRP‐conjugated secondary antibody at room temperature and washed three times with PBST. The images were scanned using the chemiluminescence detection system (Bio‐Rad). The antibodies employed were as follows: anti‐GAPDH/Gapdh (1:1000, 60004‐1; Proteintech), anti‐CDKN1A/Cdkn1a (1:1000, ab188224; Abcam), anti‐AIF/Aif (1:1000, 5318; Cell Signaling Technology). Semi‐quantification was performed with the Fiji software.

### 4.15 Statistical procedures

All assays were performed independently and in triplicate unless otherwise stated. Statistical analysis was carried out using GraphPad Prism software version 9.0 (GraphPad Software, http://www.graphpad.com, accessed in Dec 2022). The results are presented as mean values  ±  SEM. The threshold for statistical significance (p‐value) was set at .05, and statistical significance is indicated in the Figureures and legends as follows: *p ＜ .05, **p ＜ .01, ***p ＜ .001, ****p ＜ .0001, and ns indicates no significance.

## CONFLICT OF INTEREST STATEMENT

The authors declare no conflict of interest.

## Supporting information

Supporting Information

Supporting Information

Supporting Information

Supporting Information

Supporting Information

Supporting Information
