# Supplementary material for: Single‐cell landscape analysis reveals systematic senescence in mammalian Down syndrome
Source: Clin Transl Med. 2023 Jul 17;13(7):e1310. doi: 10.1002/ctm2.1310 (PMC10352595; doi:10.1002/ctm2.1310)
Supplement: Supplementary file 1 — Supporting Information [file CTM2-13-e1310-s004.docx]

Supplementary Materials for

**Single-cell Landscape Analysis Reveals Systematic Senescence in Mammalian Down Syndrome**

Yao Chen *et al.*

Corresponding authors: Dan Zhang ([zhangdan@zju.edu.cn](mailto:zhangdan@zju.edu.cn))

and Guoji Guo (ggj@zju.edu.cn)

**The PDF file includes:**

Figure. S1 to S13

Legends for Table S1 to S14

**Other Supplementary Material for this manuscript includes the following:**

Table S1 to S14


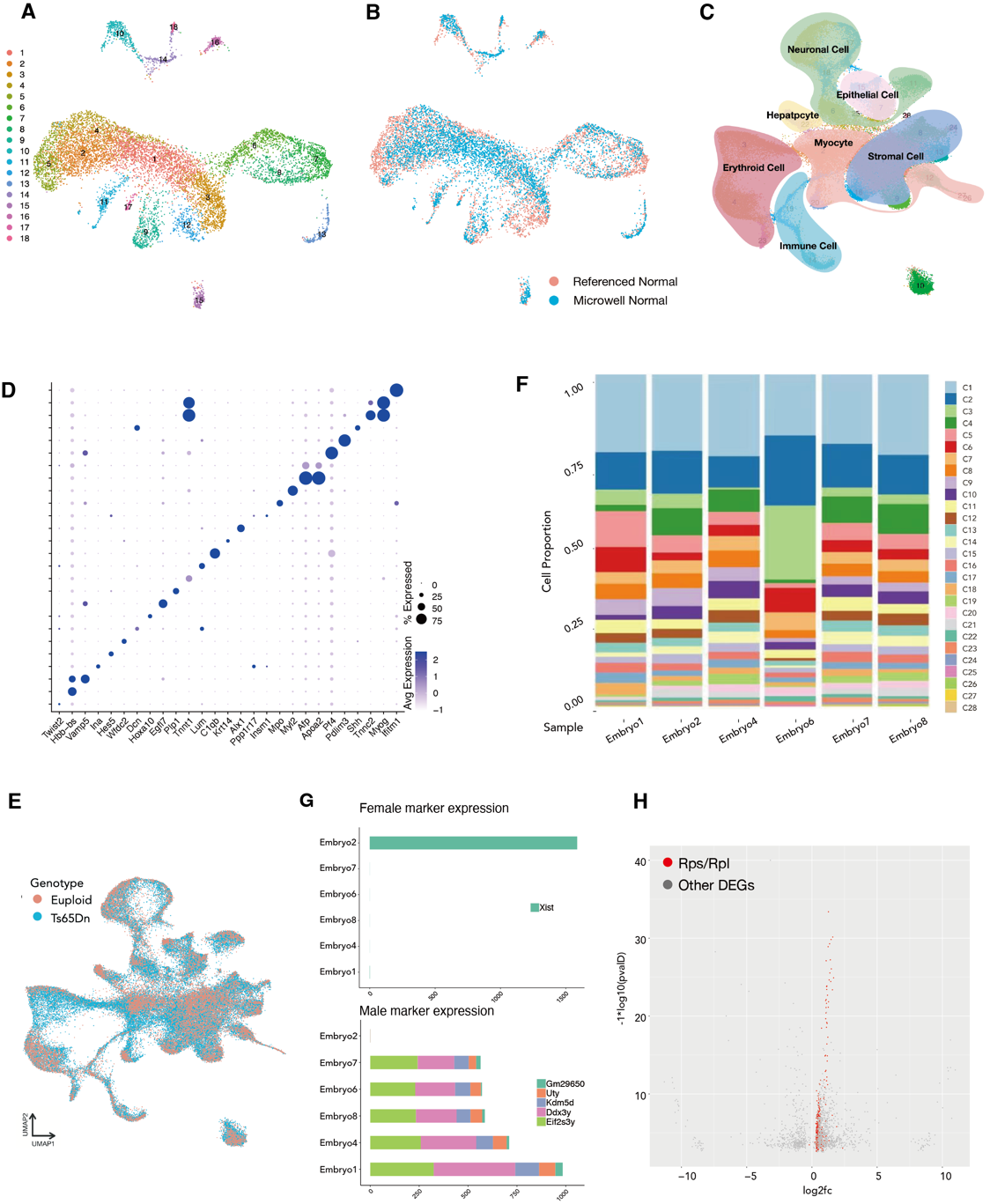
**Supplementary figure1.**

**(A)** UMAP presentation of combined referenced E11.5 mouse single-cell data with the Normal single-cell sequenced with Microwell-seq.

**(B)** UMAP presentation of combined single-cell transcriptome data from referenced E11.5 data and Microwell-seq produced Normal data. Each dot represents a single cell and is colored according to its donor of origin.

**(C)** Mouse cell lineage classification.

**(D)** Dotplot showing the expression patterns of selected marker genes of each mouse cell cluster.

**(E)** UMAP presentation of combined single-cell transcriptome data from DS and Normal mouse embryos. Each dot represents a single cell and is colored according to its donor of origin.

**(F)** The proportions of different embryos originated cells composing different cell clusters.

**(G)** Expression of sexual marker in each mouse embryo.
**(H)** Volcano plot showing DEGs between DS cells and Normal cells. Red dots represent ribosomal protein-coding genes.**
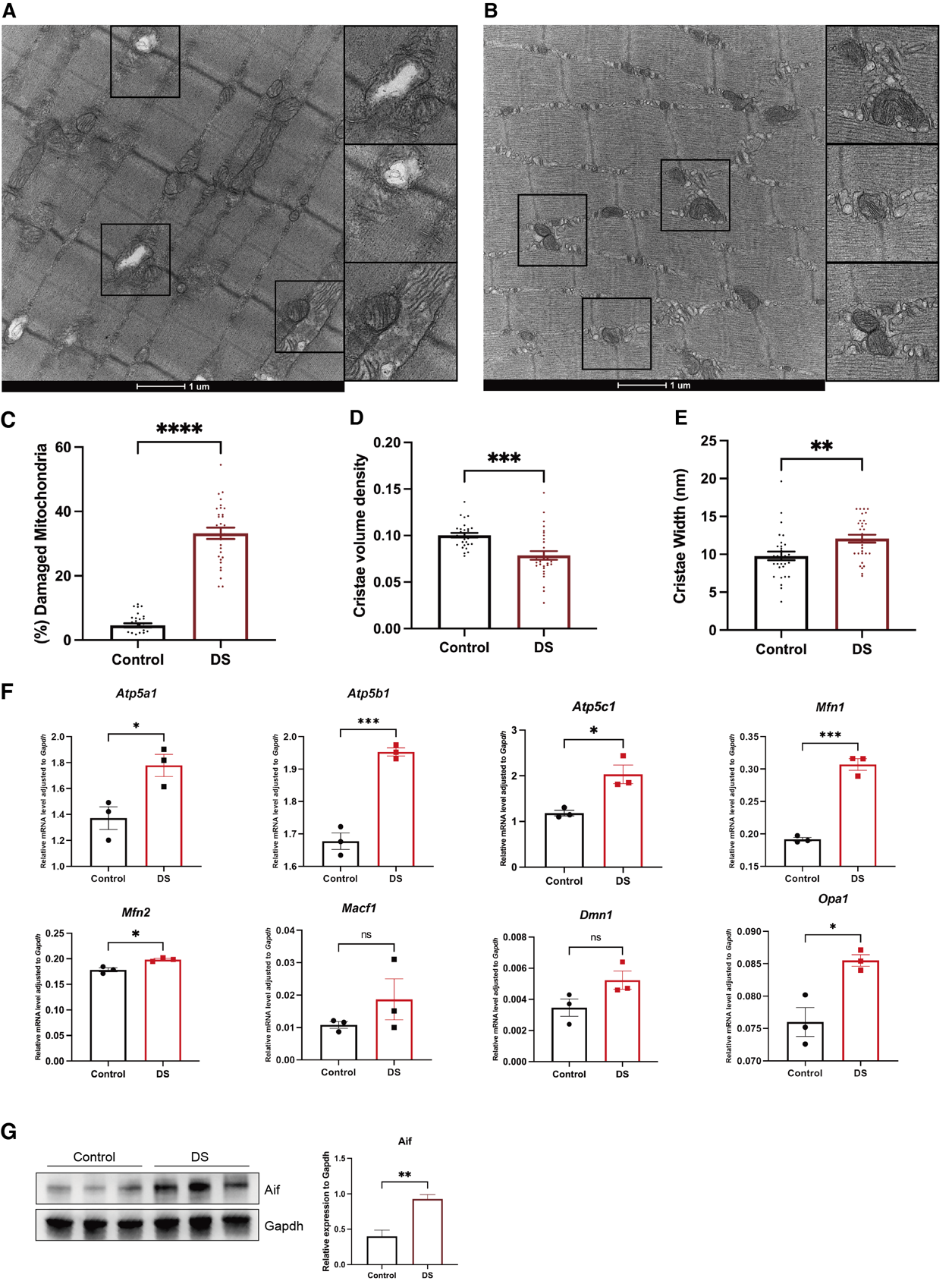
Supplementary figure2.**

**(A-B)** Representative TEM images of mouse DS (A) muscle show damaged mitochondria characterized by low electron density, while Control (B) with an intact morphology, without any particular changes in the structure.

**(C)** The percentage of damaged mitochondria is significantly higher in mouse DS muscle compared with Control muscle. Each scatter point represents non-overlapping visual fields that were randomly selected (10 fields per sample).

**(D)** To calculate cristae volume density, the cristae surface area is divided by the area of the mitochondrion. Each scatter point represents cristae volume density in one mitochondrion (10 fields per sample).

**(E)** Mean cristae maximal width in DS versus Control. Each scatter point represents cristae width (10 fields per sample).

**(F)** Relative expression levels of mitochondrial functional genes in mouse DS and normal skeletal muscle cells adjusted to those of *Gapdh*. The expression was measured by RT‒qPCR.

**(G)** Western blot analysis of mouse Aif protein expression in mouse skeletal muscle. (n=3)

**
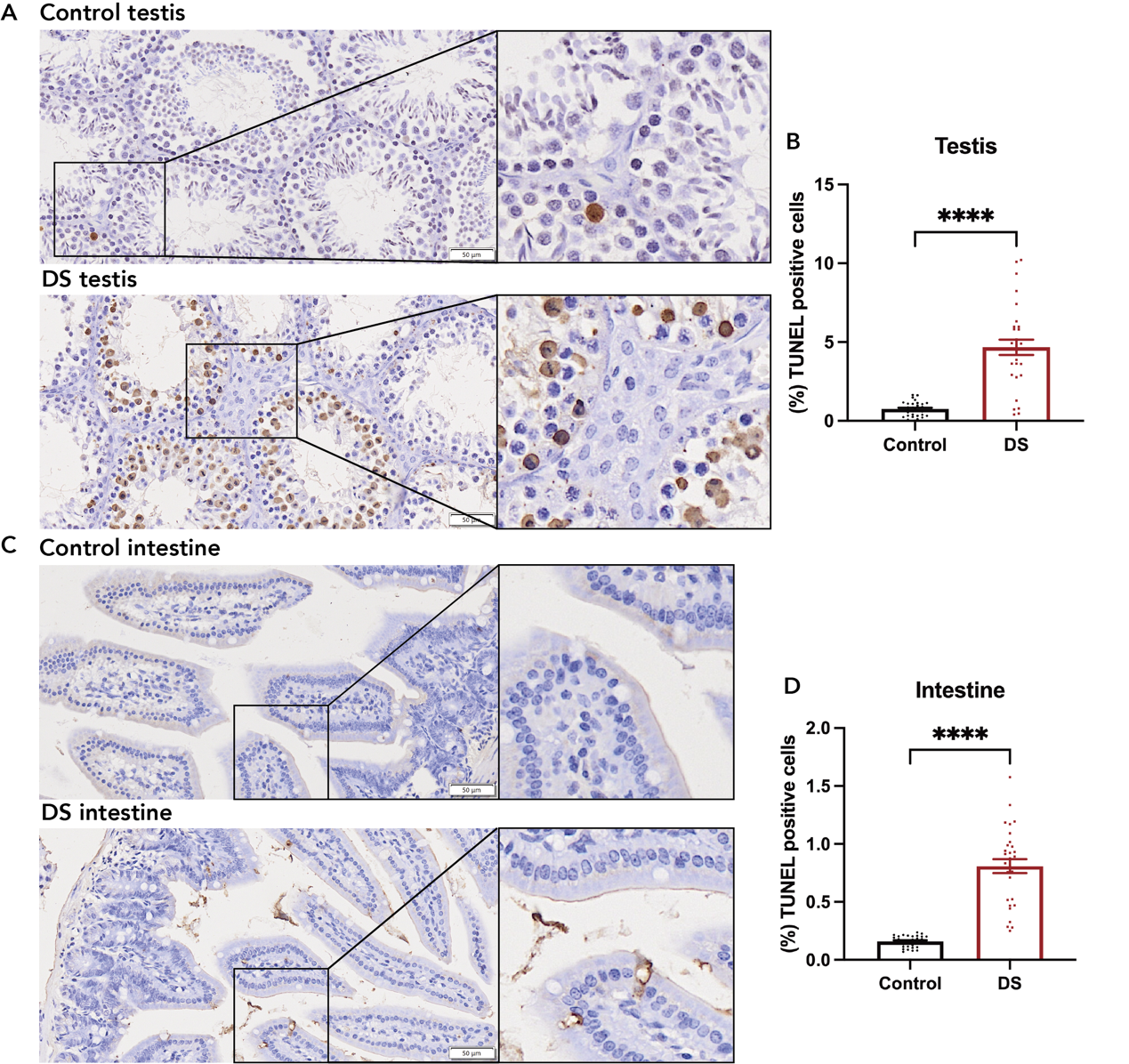
**

**Supplementary figure3.**

**(A, C)** The representative image of TUNEL staining of (3-month-old) DS and Normal mouse testis (A) and intestine (C). Bar = 50 um.

**(B, D)** Statistical analysis of TUNEL-positive cells. Each scatter point represents non-overlapping visual fields that were randomly selected (5-10 fields per sample).


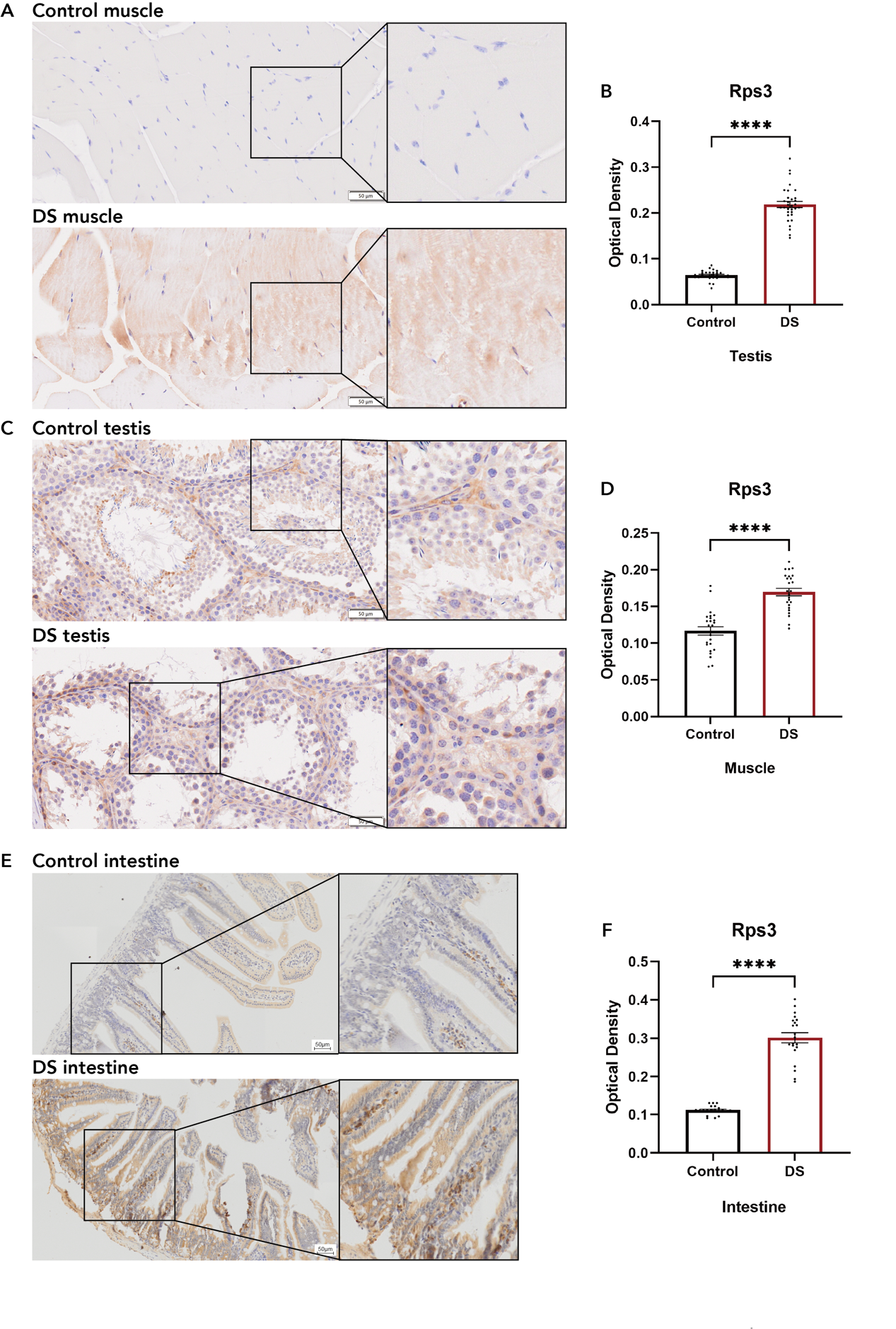
**Supplementary figure4.**

**(A, C, E)** IHC staining of Rps3 in (3-month-old) DS and Control mouse muscle (A), testis (B), and intestine (C). Bar = 50 um.

**(B, D)** Statistical analysis of the optical density of Rps3 signal. Each scatter point represents non-overlapping visual fields that were randomly selected (5-10 fields per sample).


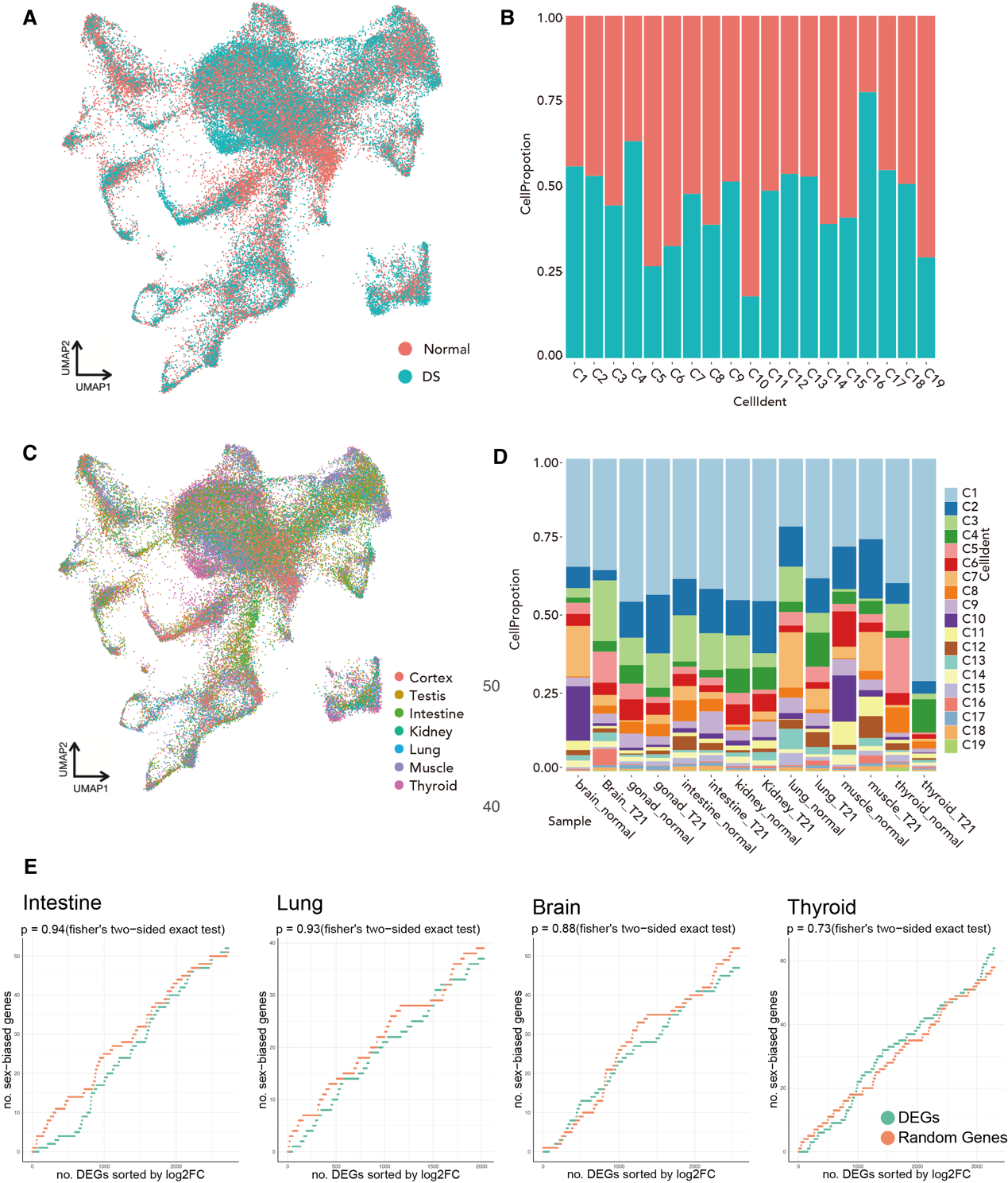
**Supplementary figure5.**

**(A)** UMAP presentation of combined single-cell transcriptome data from DS and diploid testes. Each dot represents a single cell and is colored according to its donor of origin.

**(B)** Percentage of DS and Normal cell within each cell cluster. Different colors in the inner circle stand for different major cell clusters. Different colors in the bars represent DS or Normal part as in supplementary figure 3a.

**(C)** UMAP presentation of combined single-cell transcriptome data from different tissues. Each dot represents a single cell and is colored according to its donor of origin.

**(D)** The proportions of different tissue-originated cells composing different cell clusters.

**(E)** The correlation of the tissue-specific sexual bias genes and randomly selected DEGs.


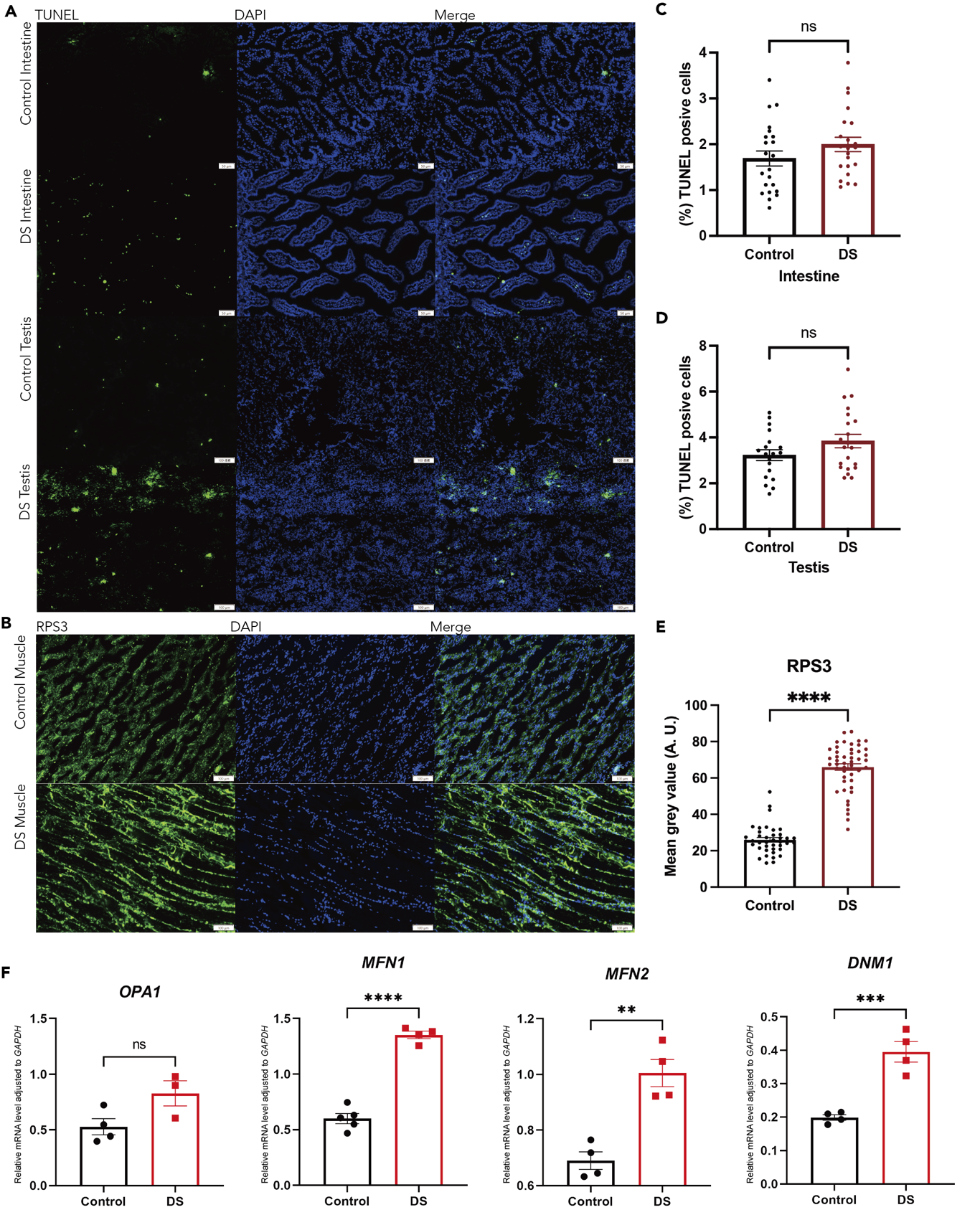


**Supplementary figure6.**

**(A)** Representing IF staining images of TUNEL in 20GW human fetal intestine (Line 1, 2) and testis (Line 3, 4). Bar = 100 um.

**(B)** Representing IF staining images of RPS3 in 20GW human fetal muscle. Bar = 100 um.

**(C, D)** Statistical analysis of the percent of TUNEL-positive cells. Each scatter point represents non-overlapping visual fields that were randomly selected; one Control sample vs one DS sample; n (fields)＞20 per sample.

**(E)** Statistical analysis of the percent of RPS3 staining. Each scatter point represents non-overlapping visual fields that were randomly selected; three Control samples vs three DS samples; n (fields) = 5 per sample.

**(F)** Relative expression levels of mitochondrial functional genes in human fetal DS and normal skeletal muscle cells adjusted to those of GAPDH. The expression was measured by RT‒qPCR.

The bars show mean values ± SEM.


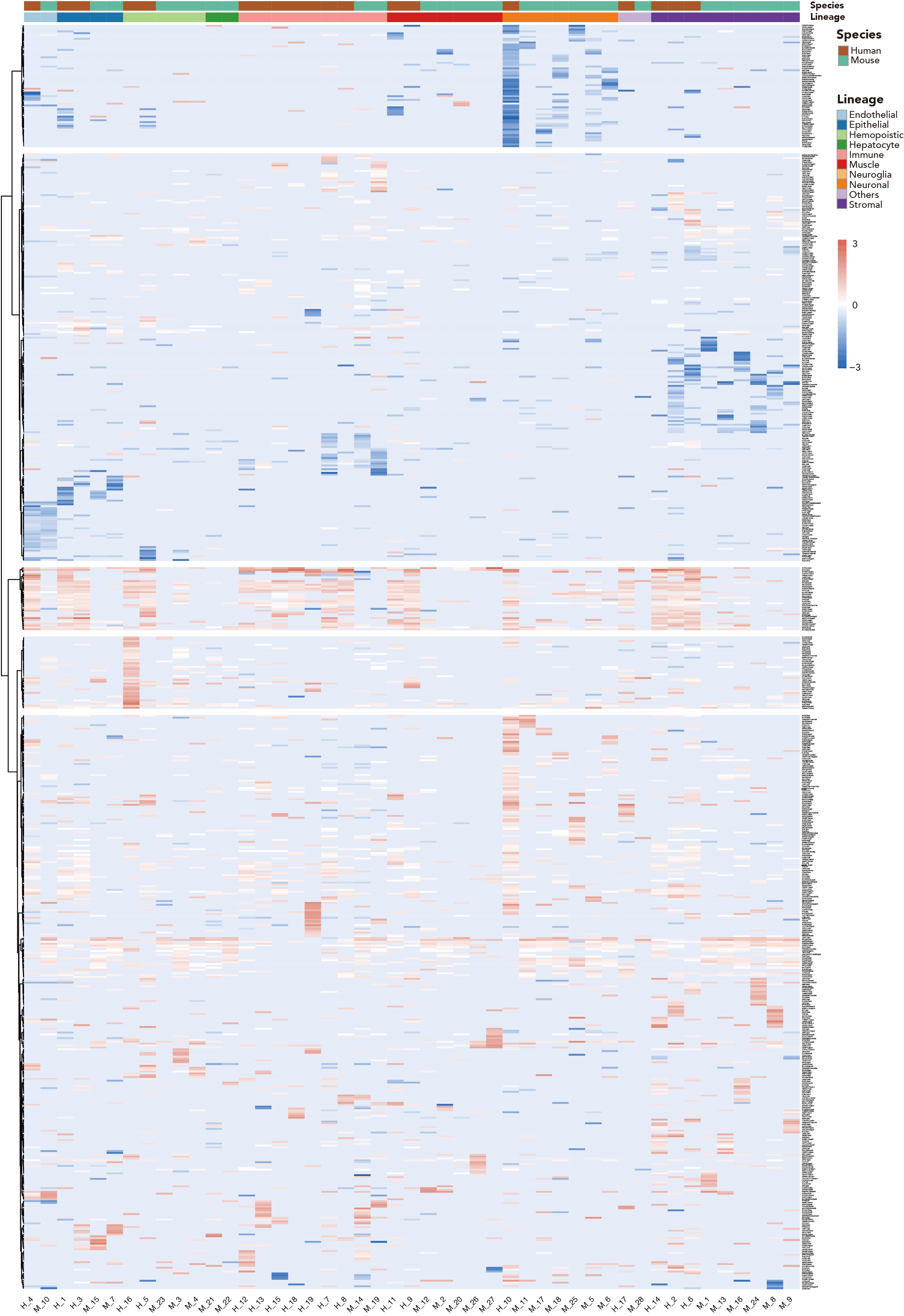


**Supplementary figure7.** The fold change of homologous DEGs across human and mouse datasets. The color shades represent the fold of change.


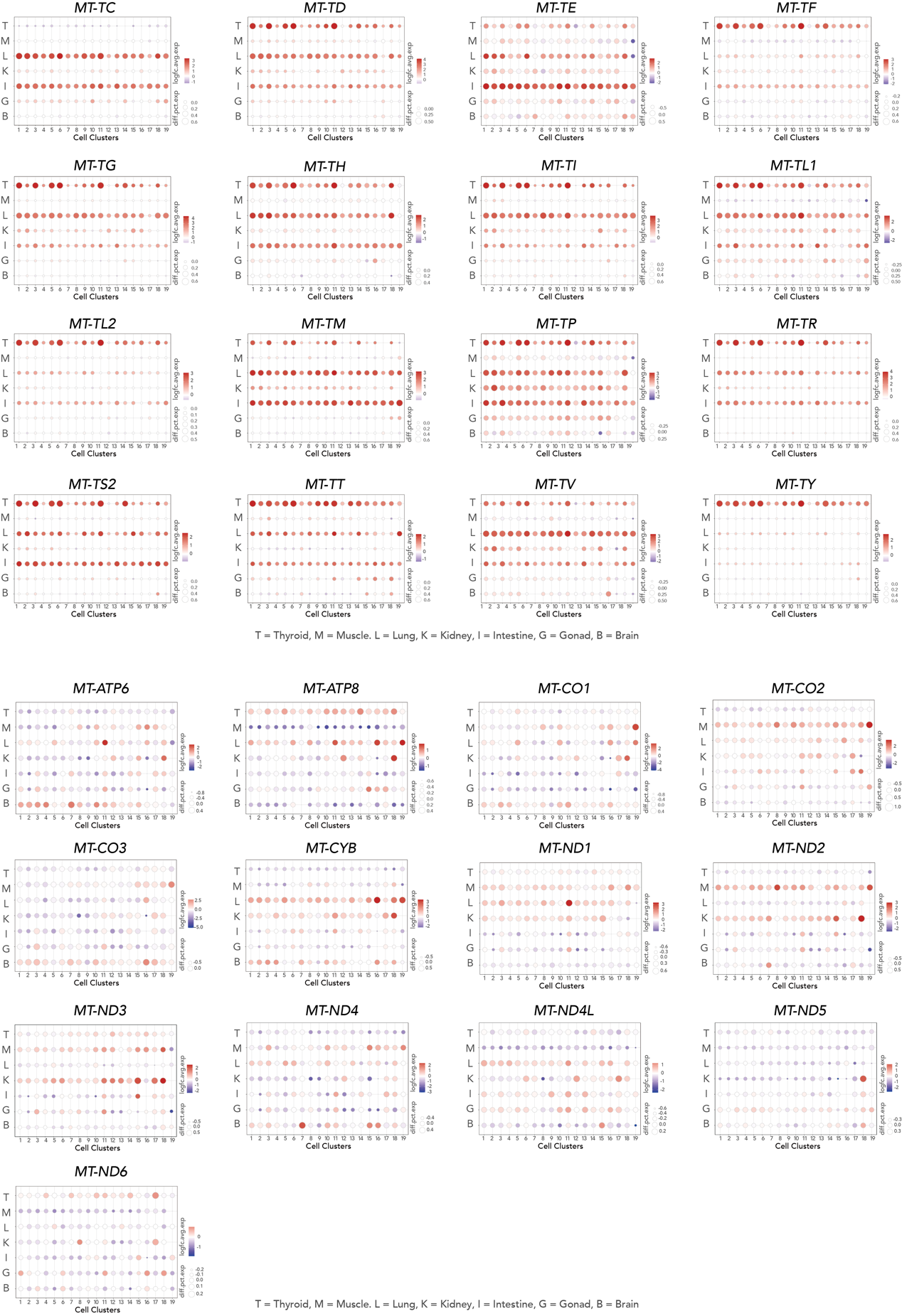


**Supplementary figure8.** Dot plot visualization of the relative expression level of the mitochondrial tRNA and mRNA in DS cells compared to Normal cells. The size of the dot encodes the percentage of cells within a cell cluster, and the color encodes the average expression level. Columns represent cell clusters (C1-C19), rows represent different organs (T = Thyroid, M = Muscle, L = Lung, K= Kidney, I = Intestine, G = Gonad, B = Brain).


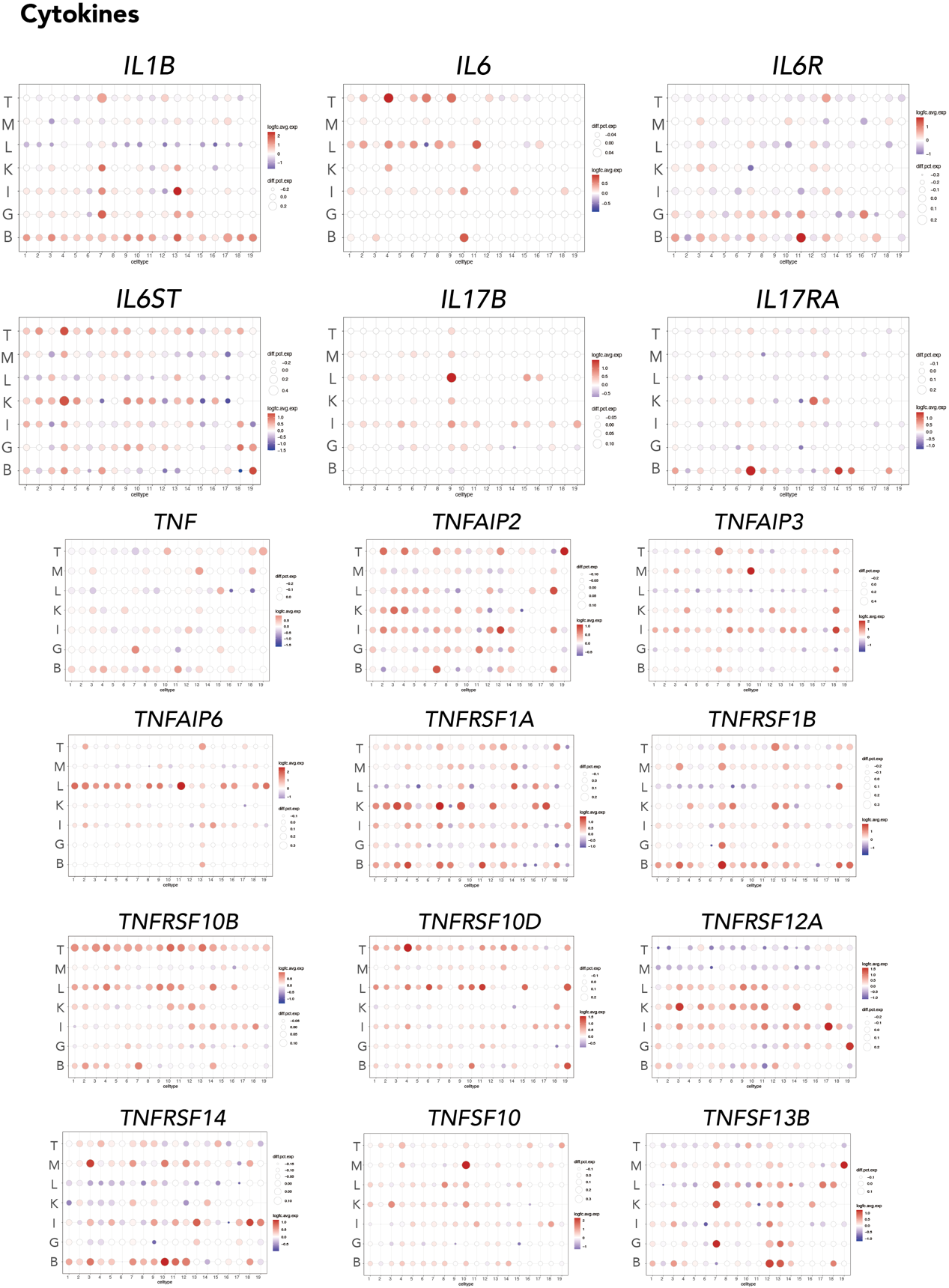

**Supplementary figure9.** Dot plot visualization of the relative expression level of the cellular senescence-associated genes in DS cells compared to Normal cells. The size of the dot encodes the percentage of cells within a cell cluster, and the color encodes the average expression level. Columns represent cell clusters (C1-C19), rows represent different organs (T = Thyroid, M = Muscle, L = Lung, K= Kidney, I = Intestine, G = Gonad, B = Brain).

**
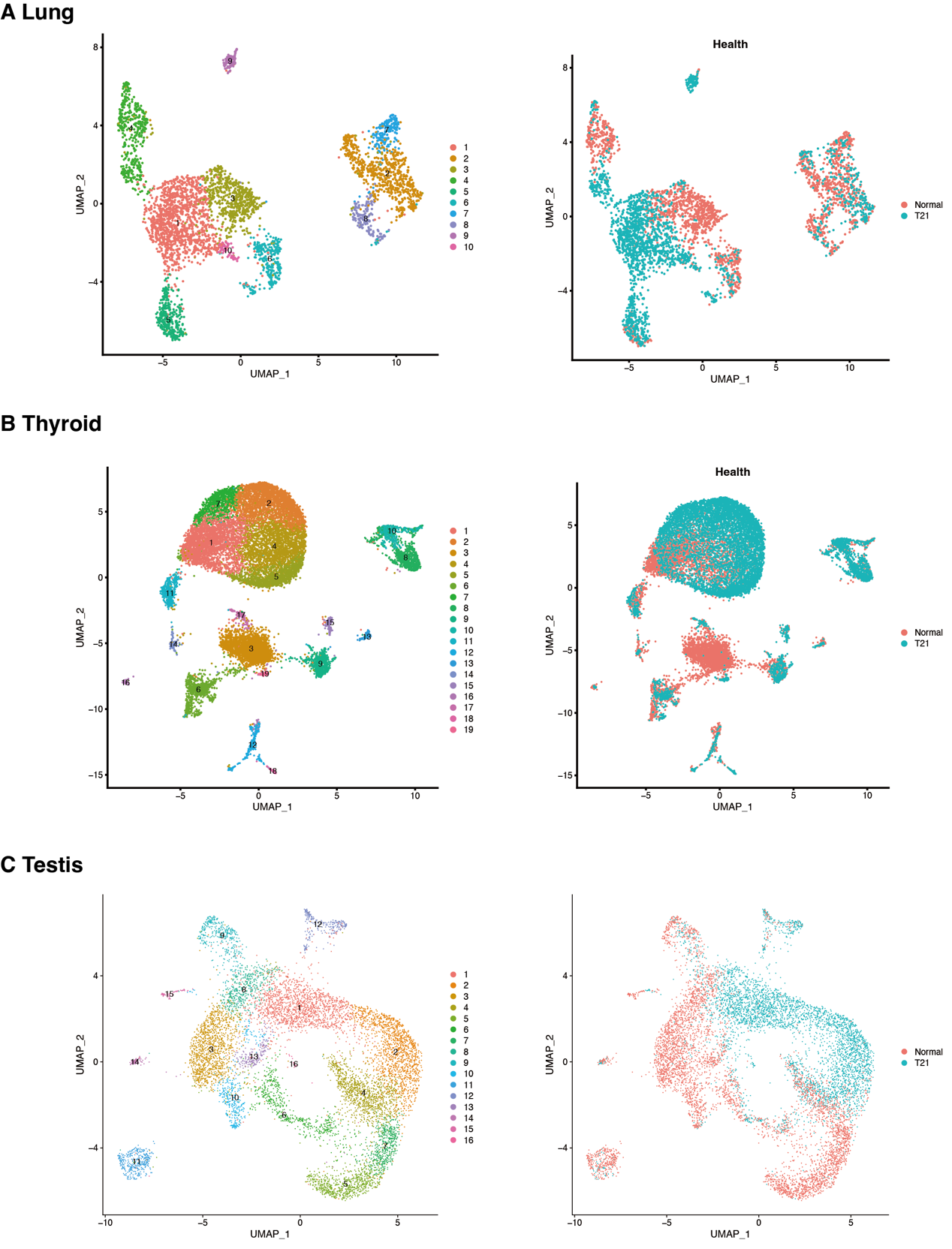
**

**
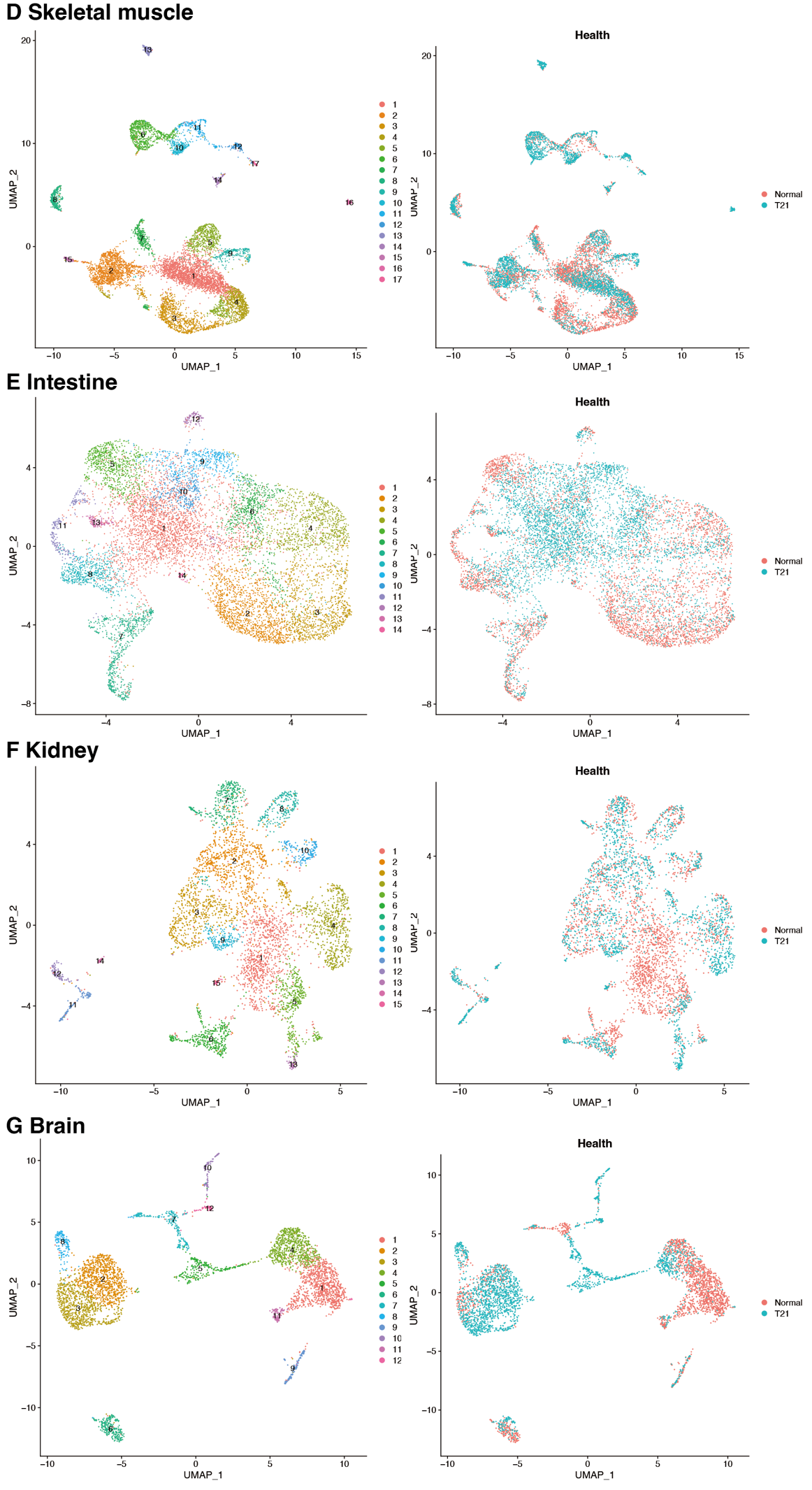
**

**Supplementary figure10.** UMAP presentation of each human organ. Cell clusters were labeled in different colors (left) and karyotypes (right).


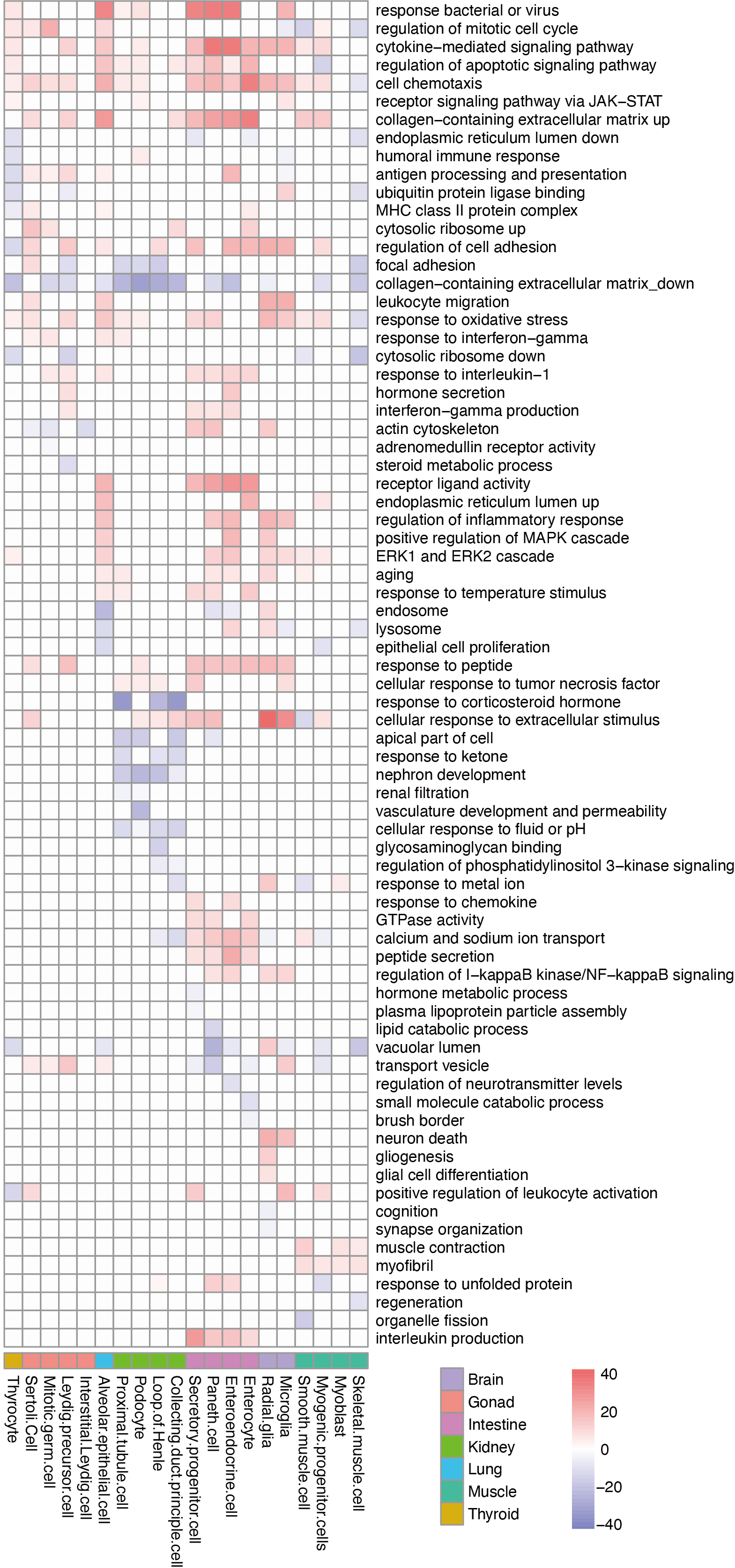
**Supplementary figure11.** GO of subcluster-specific differential expression, colored by gene number. The red square represents the up-regulated GO terms, while the green represents down.


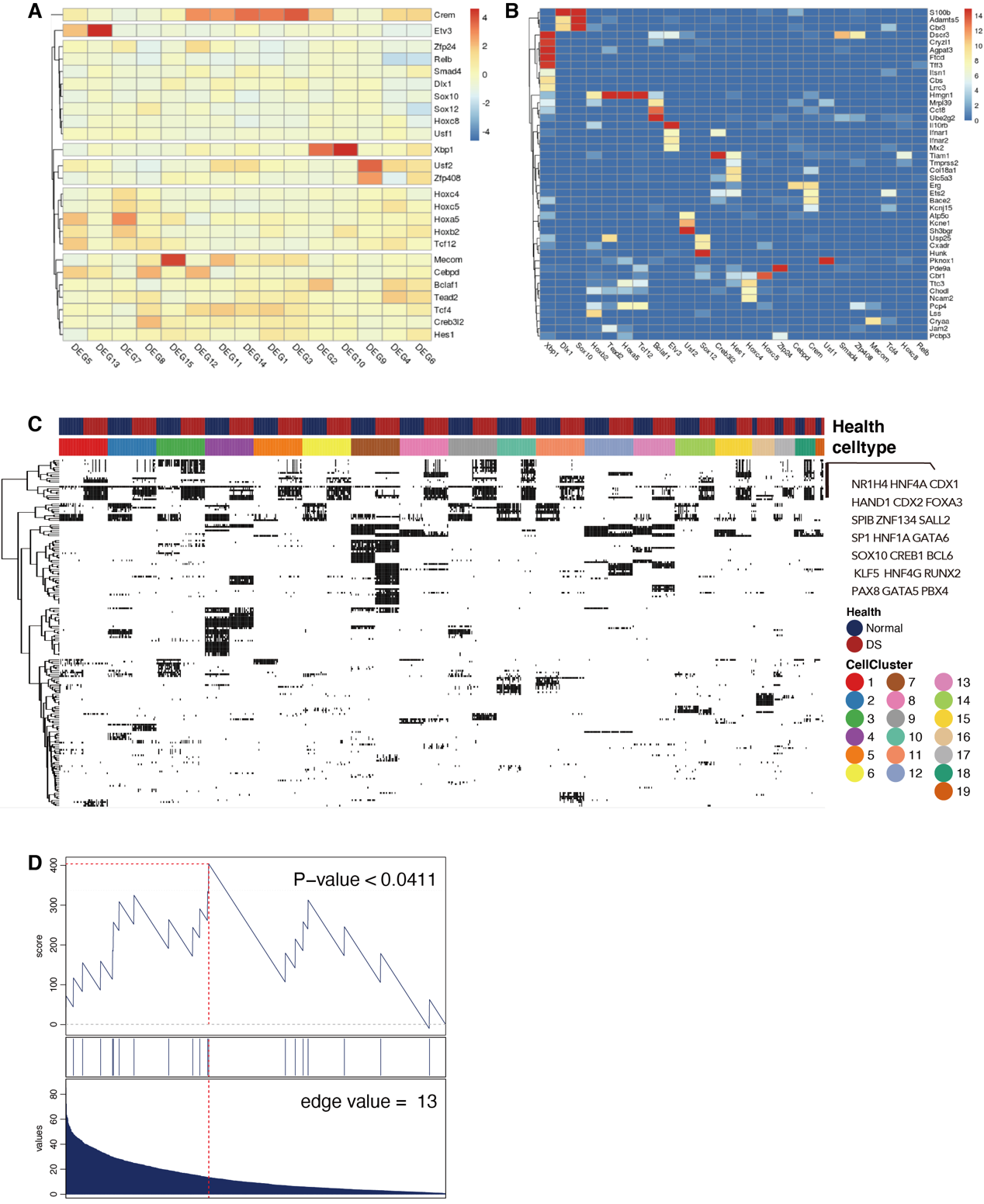
**Supplementary figure12. (A)** The correlation of the SCENIC-identified TFs was identified with each of the mouse DEG clusters. **(B)** The correlation of HSA21 (homologous) genes with active TFs. **(C)** Heatmap of the binarized TF regulatory networks identified using SCENIC. **(D)** GSEA plot for the enrichment of the TFs widely upregulated in human DS based on the hypergeometric distribution of the largest weight of links between a TF and all HSA21 genes.


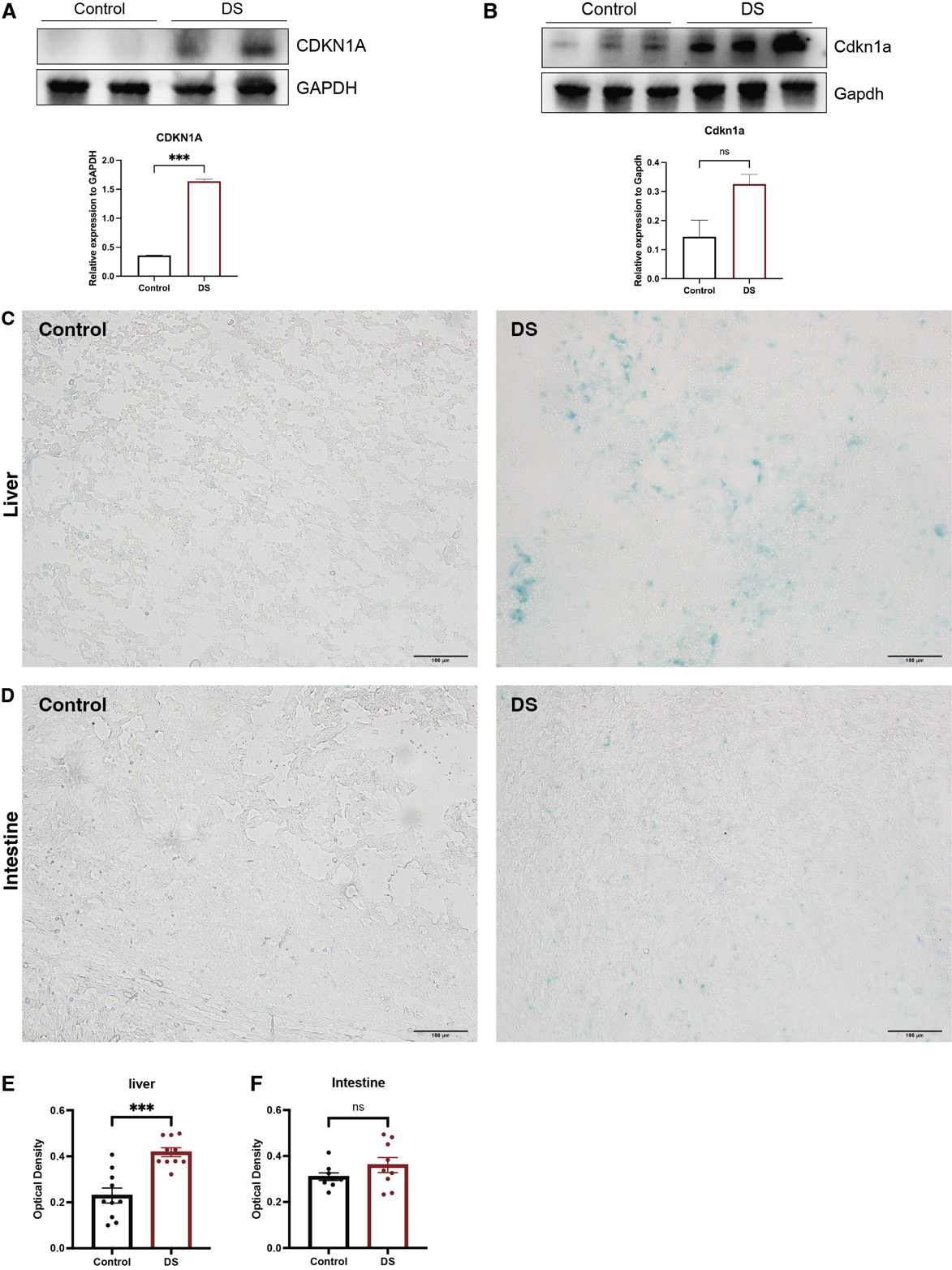
**Supplementary figure13.**

**(A, B)** Western blot analysis of human CDKN1A (A) and mouse Cdkn1a (B) protein expression in both groups. (n=2-3)

**(C, D)** The representative image of SA-β-GAL staining of the human fetal liver (C) and intestine (D). Bar = 100 um.

**(E, F)** Statistical analysis of SA-β-GAL staining signal. Each scatter point represents a randomly selected slide; three Control samples vs three DS samples; n=3 per sample.

**Legends for data files S1 to S14**

**Table S1.** Differentially expressed genes detected in 28 mouse cell clusters

**Table S2.** Differentially expressed genes detected in each of the mouse cell clusters

**Table S3.** Differentially expressed genes classified into 15 mouse DEG clusters

**Table S4.** Differentially expressed genes detected in 19 human cell clusters

**Table S5.** Differentially expressed genes detected in each of the human cell clusters, tissue information attached

**Table S6.** Homologous differentially expressed genes detected in human and mouse

**Table S7.** Differentially expressed genes classified into 15 human DEG clusters

**Table S8.** Differentially expressed genes detected in clusters of different human fetal organs, sheets spread by organs

**Table S9.** Differentially expressed genes detected in each of the cell clusters, sheets spread by organs

**Table S10.** GO terms of the up-and down-regulated DEGs by clusters, sheets spread by organs

**Table S11.** Transcription factors ordered in the human and mouse SCENIC heatmap

**Table S12.** Intersect genes between DS cell clusters and referred senescence models

**Table S13.** Primer information of rt qPCR targets

**Table S14.** Basic information about samples been processed
